# Toward Grid-Based Models for Molecular Association

**DOI:** 10.1021/acs.jctc.4c01293

**Published:** 2025-01-13

**Authors:** Hana Zupan, Bettina G. Keller

**Affiliations:** Department of Biology, Chemistry and Pharmacy, Freie Universität Berlin, Arnimallee 22, 14195 Berlin, Germany

## Abstract

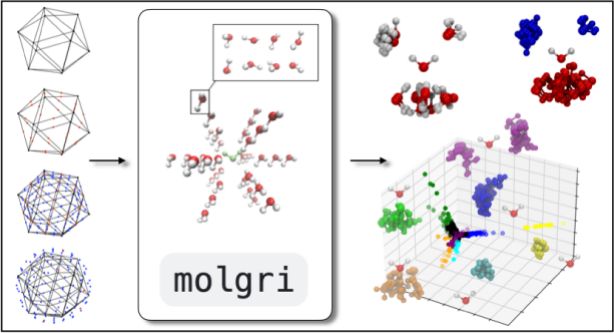

This paper presents a grid-based approach to model molecular association processes as an alternative to sampling-based Markov models. Our method discretizes the six-dimensional space of relative translation and orientation into grid cells. By discretizing the Fokker–Planck operator governing the system dynamics via the square-root approximation, we derive analytical expressions for the transition rate constants between grid cells. These expressions depend on geometric properties of the grid, such as the cell surface area and volume, which we provide. In addition, one needs only the molecular energy at the grid cell center, circumventing the need for extensive MD simulations and reducing the number of energy evaluations to the number of grid cells. The resulting rate matrix is closely related to the Markov state model transition matrix, offering insights into metastable states and association kinetics. We validate the accuracy of the model in identifying metastable states and binding mechanisms, though improvements are necessary to address limitations like ignoring bulk transitions and anisotropic rotational diffusion. The flexibility of this grid-based method makes it applicable to a variety of molecular systems and energy functions, including those derived from quantum mechanical calculations. The software package MolGri, which implements this approach, offers a systematic and computationally efficient tool for studying molecular association processes.

## 1 Introduction

The vastness of configuration space is a fundamental problem in molecular dynamics (MD) simulations. Due to its immense dimensionality and the fact that only narrow regions are significantly populated, often separated by large barriers, obtaining a comprehensive view of likely configurations and their transition time scales is clearly challenging. MD simulations explore this space by taking steps (determined by Newtonian forces and the choice of a thermostat) in configuration space that involve small changes in all degrees of freedom (DoF) at once. If the simulation is ergodic, every region of space will be visited proportionally to its Boltzmann weight, given infinite simulation time. However, even if ergodicity is formally fulfilled, there is no guarantee that all regions of interest have been sufficiently sampled within the finite time of a simulation. It is difficult to even determine whether all low-energy states have been reached.^1,2^

Markov state models (MSMs)^3−6^ are a powerful tool to analyze complex molecular dynamics. They reduce the complexity of the high-dimensional, continuous dynamics by discretizing the configurational space into grid cells. The system’s dynamics are then modeled as transitions between these grid cells, where the transition probability is estimated from MD simulations. The resulting transition probability matrix allows for a quantitative analysis of the molecular dynamics in terms of metastable states, mean-first passage times and pathways between different regions of configurational space. MSMs thus give insight into the mechanism of multistate molecular dynamics. One of the fields in which MSM have been particularly useful is in modeling molecular association, such as protein–ligand and protein–protein binding.^7−9^ With recent advances in electronic structure methods^10,11^ and the advent of neural network potentials,^12,13^ it is likely that MSMs will be applied to more diverse molecular associations processes, such as adsorption on surfaces, the formation of nanoparticles or encounter complexes of chemical reactions.

However, MSMs, in particular MSMs of molecular association processes, are very sensitive to statistical uncertainties^14,15^ and therefore often require extensive MD simulations. Approaches to improve the statistical efficiency of MSM estimations include improved feature selection for the definition of the underlying grid,^16,17^ variational and core-set Markov models,^4,18,19^ adaptive sampling algorithms,^20^ and enhanced sampling combined with dynamical reweighting.^21^ Despite these advances, MSM studies remain subject to the assumption that statistical noise does not distort the results.

An alternative is a generative grid-based approach, which we are pursuing in this contribution. The idea is to systematically produce structures at selected grid points in configuration space, calculate the point energies of generated structures and use this information along with the geometrical properties of the grid cells to obtain a probability flow across the cell boundaries. From these probability flows, one can calculate the transition rate matrix, a close analogue to the MSM transition probability matrix. Thus, instead of extensive MD simulations, only a single energy calculations per grid-point is needed. In addition, this approach guarantees that all regions of space are taken into account up to the boundaries and the resolution of the grid.

The grid-based approach, including the formula for the pairwise transition-rate constants, is derived^22−25^ by assuming that the system evolves according to overdamped Langevin dynamics in a collective variable space and by discretizing the associated Fokker–Planck operator, leading to the square-root approximation of the Fokker–Planck operator (SqRA). The method has shown excellent replication of sampling-based MSMs for low-dimensional Cartesian spaces.^22,23,25,26^ A proof of principle for alanine-dipeptide has been reported in ref (23). However, a crucial assumption in the SqRA is that the grid cells are so small that the potential within each grid cell is essentially constant. Thus, grids with high resolution are required, effectively limiting the grid-based approach to low-dimensional collective variable spaces.

Here, we consider the association of two molecules *A* and *B* in solution. The formation of bimolecular complexes typically consists of two stages: 1) diffusion-based association and 2) interaction-based completion of binding.^27^ Comprehensive sampling of the diffusion-based association is almost intractable with standard molecular simulation as the simulation time needed to explore all possible relative translations and relative orientations of the two molecules is immense. However, within the rigid-body approximation, this process reduces to diffusion in the six-dimensional space of translation and rotation of molecule *B* relative to molecule *A*.

There are two major challenges in constructing translational and rotational grids for a SqRA-Markov model. First, the grid must be uniform, meaning that all grid cells should have approximately the same size. Second, it is necessary to calculate both the six-dimensional volume of each grid cell and the five-dimensional hypersurface area that represents the boundary between neighboring cells. In ref^28^ we benchmarked methods for constructing uniform grids in translational space. Here, we extend the discretization to the full six-dimensional translation and rotation space. Drawing inspiration from the robotics community,^29−31^ we employ regular Voronoi tessellation of the rotational space using quaternions, and we provide equations for the corresponding grid cell volumes and surfaces. We have developed a Python package, MolGri, which generates grids for the six-dimensional translation and orientation space, calculates the geometric parameters of the grid, and interfaces with MD programs to obtain the grid energies and compute the rate matrix. At the current stage, the package does not yet account for transitions into the bulk and for anisotropic rotational diffusion. We discuss the remaining steps needed to achieve an accurate SqRA-Markov model for molecular association processes.

## 2 Theory

### 2.1 Square-Root Approximation

The square-root approximation has been derived and tested in refs.^22−25^ In this section, we summarize the most important equations. We additionally provide a more detailed derivation in the Supporting Information.

Consider a molecular system with *N* atoms and 3*N* translational degrees of freedom. A collective variable *x*_*i*_ is a function that maps the 3*N* translational degrees of freedom to a real number: . We assume that in a low dimensional collective variable space **x** = (*x*_1_, *x*_2_, ..., *x*_*m*_) ∈ Ω ⊂ , where *m* ≪ 3*N*, the dynamics of the system can be modeled by overdamped Langevin dynamics:
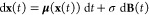
1where **B**(*t*) = (*B*_1_(*t*) ... *B*_m_(*t*)) is an *m*-dimensional Wiener process, **μ**(**x**(*t*)) = −ξ^–1^*M*^–1^∇*V*_*eff*_(**x**(*t*)) is the *m*-dimensional drift vector, and  scales the Wiener process and is linked to the diffusion of the system in the collective variable space. We assume that the diffusion is isotropic in the collective variable space, and hence σ is simply a scalar. For nonisotropic diffusion, σ has to be replaced by an (*m* × *m*)-matrix. ξ is a friction parameter with units s^–1^, *M* is the effective mass,  is the effective potential in the collective variable space, *k*_*B*_ is the Boltzmann constant, *T* is the temperature, and∇*f*(**x**) denotes the gradient of a function .

*ρ*(**x**, *t*) is a probability density in the space of collective variables, whose time-evolution is governed by the Fokker–Planck equation associated with eq 1,
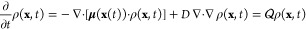
2where  is the Fokker–Planck operator. For a vector field , ∇ · **f**(**x**) denotes the divergence of the vector field. *D* = *σ*^2^/2 = *k*_B_*Tξ*^–1^*M*^–1^ is the diffusion constant. The stationary density associated with eq 2 is the Boltzmann density
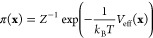
3where  is the configurational partition function.

The collective variable space Ω is discretized into *N*_*d*_ nonoverlapping grid cells , where **x**_*α*_ denotes the center of cell Ω_*α*_. On this grid, eq 2 can be approximated by a matrix-vector equation
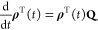
4where the *N*_*d*_-dimensional vector  contains the time-dependent probabilities to find the system within each grid cell, and
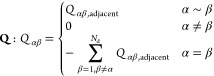
5is a rate matrix and the discretized version of the Fokker–Planck operator . *α* ∼ *β* indicates that Ω_*α*_ and Ω_*β*_ are adjacent. The rate matrix **Q** is related to the MSM transition matrix **P**(*τ*_MSM_) by^21^
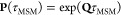
6where *τ*_MSM_ is the MSM lag time.

The square-root approximation of ,^22,23,25^ provides an analytical expression for the transition rate constant between adjacent cells
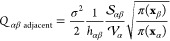
7where h_*αβ*_ = |**x**_*β*_ – **x**_*α*_| is the Euclidean distance between two cell centers.  is the volume of Ω*_α_*, and  is the surface area of the intersecting (hyper-)surface between adjacent cells Ω*_α_* and Ω_*β*_. eq 7 relies on the following assumptions:

1 The grid is a Voronoi grid.
2 The grid cells are small, so that *V*_eff_(**x**), *π*(**x**), and *ρ*(**x**) are approximately constant within a grid cell.
3 Diffusion is isotropic, so that *σ* = const.

The significance of eq 7 is that, given the geometric parameters of the grid *h*_*αβ*_,  and , along with the effective potential energy at the grid cell centers *V*_eff_(**x**_*α*_), one can construct an MSM without the need for MD simulations.^22,23,25^

Note that, in eq 5, we ensured that the row-sum of the rate matrix is zero. This convention is consistent with the MSM convention, in which the transition matrix is usually row-normalized to one. However, in communities that work with rate matrices rather than transition matrices, by convention, the columns of the rate matrices are normalized to zero.^21^ This yields the transpose of **Q**.

### 2.2 Rigid Body Approximation and Coordinate System

To model molecular association, we consider a molecular system with two molecules *A* and *B* in the absence of any external potential. Since the total energy is independent of the overall translation and rotation of the system, we can reconceptualize it as the molecule *A* completely fixed at origin and the molecule *B* free to translate (3 DoF) and rotate (3 DoF) as a rigid body. We therefore choose the molecular frame of molecule *A* as a our coordinate system, i.e., the Cartesian coordinate frame whose origin is at the center of mass of molecule *A* and whose three axes are aligned with the principal axes of inertia of molecule *A*. The Cartesian coordinates of the two molecules in this coordinate system are denoted as
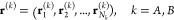
8where *N*_*A*_ and *N*_*B*_ are the respective numbers of atoms, and  is the position of the *i*th atom in the respective molecule. The vector **r**^(*k*)^ can also be represented by translational, rotational and internal coordinates
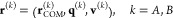
9The 3-dimensional center-of-mass coordinate is
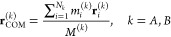
10where  is the mass of the *i*th atom, and  is the total mass of the respective molecule. It describes the translation of the respective molecule with respect to the origin of the coordinate system. Hence, . To construct the translation grid, we describe the center-mass-coordinate of *B* in spherical coordinates **r**_COM_ = (*r*, θ, ϕ) ∈ , where  is the radius, *θ* ∈ [0, *π*] is the polar angle, and *ϕ* ∈ [0, 2*π*] is the azimuth angle. The angles (*θ*, *ϕ*) ∈ *S*^2^ define a point on the three-dimensional unit sphere (2-sphere). As a product, the radius and two angles cover the 3-dimensional space: .

**q**^(k)^ ∈ *SO* (3) represents the three rotational degrees of molecule *k* = *A*, *B* with respect to a reference rotation . *SO*(3) is the rotational group and *R*(**q**^(*k*)^) is the rotation matrix that transforms  into **q**^(*k*)^. We use unit quaternions **q** = (*q*_0_, *q*_1_, *q*_2_, *q*_3_), ∥**q**∥_2_ = 1, to describe the rotation of the molecule. See ref (32) for a review on different representations of the rotational group *SO*(3) and ref (33) for more information on quaternions. Unit quaternions cover a 3-sphere (unit hypersphere) **q** ∈ *S*^3^. Each quaternion **q** corresponds to a rotation *R*(**q**) in three-dimensional space.^34^ However, each rotation in three-dimensional space is represented by exactly two quaternions, because the rotation induced by **q** equals that of −**q**: *R*(**q**) = *R*(−**q**). The relationship of quaternion **q** to a 3 × 3-rotation matrix *R*(**q**) can be expressed as^35^


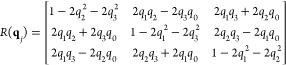
11To avoid the double coverage *R*(**q**) = *R*(−**q**) we always select one out of the quaternion pair by limiting ourselves to , where  denotes the “upper half” of the hypersphere to describe a rotation. (Select quaternions with q_0_ > 0. For *q*_0_ = 0, quaternions with q_1_ > 0 are included. If *q*_1_ = 0, the decision is based on the third component.)

Finally,  are the 3*N*_*k*_ – 6 internal degrees of freedom. Within the rigid-body approximation, we assume that the internal degrees of freedom are constant: **v**^(*k*)^ = const with *k* = *A,B*. Because we aligned the coordinate system with the molecular frame of molecule *A*, its translation and rotation also remain constant. Thus, within this model, the dynamics of the system is given by changes in  and . The collective variable vector in eq 1 then is
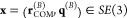
12where  is the special Euclidean group and describes the complete configuration space of the rigid body motion.

### 2.3 Translation and Rotation Grid

To systematically generate configurations of *B* relative to *A*, we discretize *SE*(3) by constructing grids for the translation and rotation subspaces  and *SO*(3). This involves constructing uniform grids on  as well as (hyper)spheres *S*^2^ (translation space) and *S*^3^ (rotation space), both of which are closely related rotation group *SO*(3). This is a challenging task.^33,36−38^ In ref (28) we compared several algorithms and concluded that a polyhedron/polytope approach for grids on *S*^*n*^ fits our needs best.

**Figure 1:**
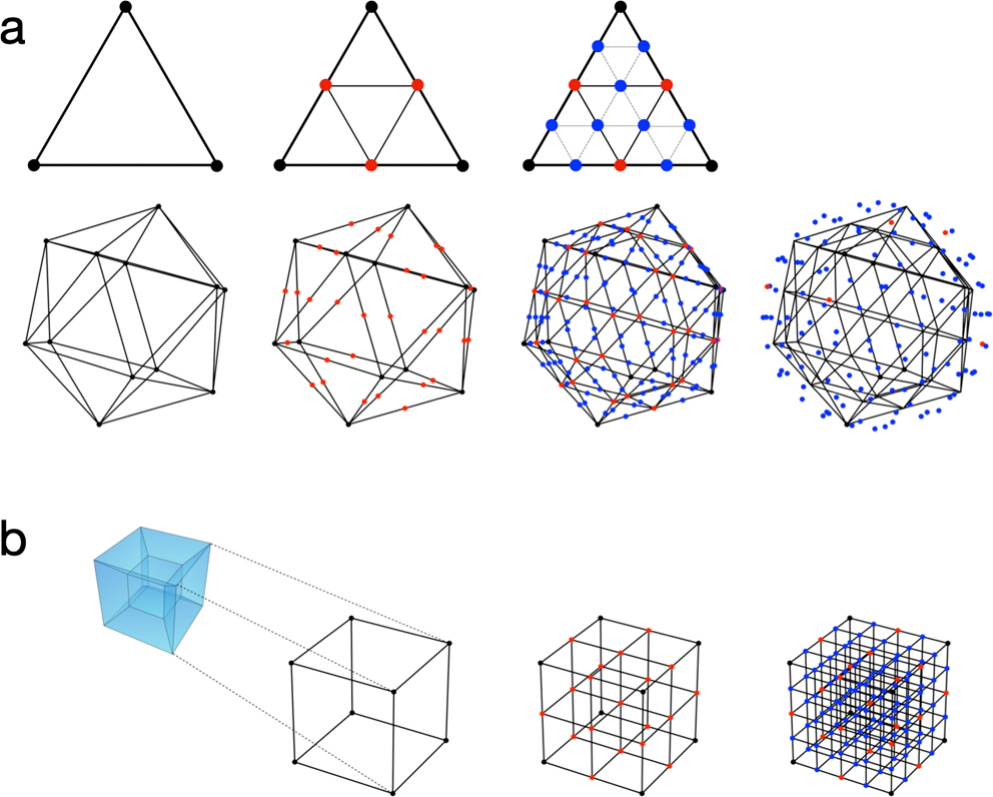
Illustration of grid construction for *S*^2^ (**a**) and *S*^3^ (**b**). For *S*^3^, only one of hypercube cells is shown, as the projection onto the 3-sphere cannot be depicted.

**Figure 2:**
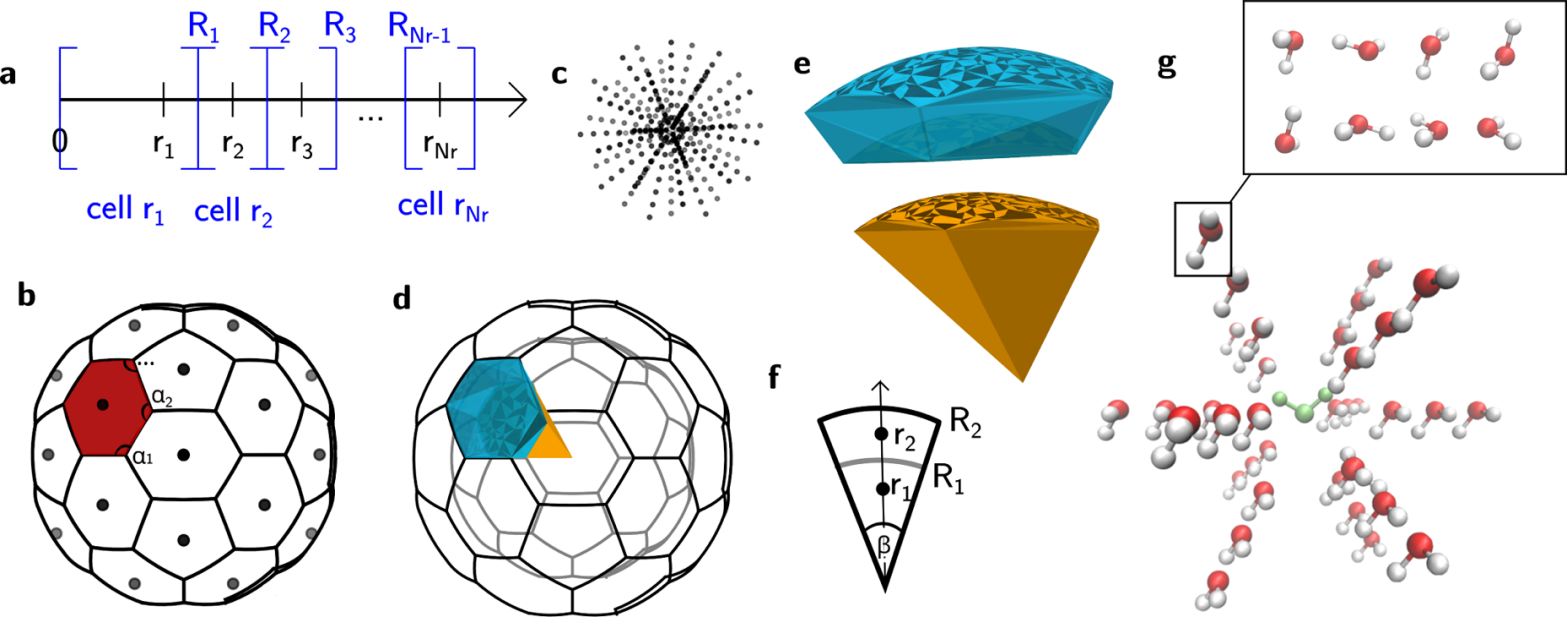
Translation grid. **a** radial grid with blue lines showing the cell boundaries. **b** angular grid, with spherical Voronoi division of the unit sphere (example of 42-point icosahedron grid), area shaded in red denotes the cell assigned to this grid point. **c** Example of translation grid with *N*_*s*_ = 42 and *N*_*r*_ = 7. **d** Partition of translation space into cells (only two radial layers are shown for clarity). The volumes of two cells are shown in color. **e** Close-up of the two colored cells of translation grid. **f** Schematic view of side borders between cells. **g** Grid with *N*_*r*_ = 3, *N*_*s*_ = 12 and *N*_*o*_ = 8 points applied to the system of two water molecules. All molecular translations generated with this grid are shown and the eight orientations are shown just for one example. The stationary molecule *A* is shown in green.

For the translation grid, we discretize radius  and angles (*θ*, *φ*) ∈ *S*^2^ separately. A uniform grid on  is straightforward: radial grid points *r*_*i*_, *i* = 1,2...*N*_*r*_, are equidistantly spaced between selected *r*_min_ and *r*_max_. The polyhedron approach to discretize *S*^2^ is illustrated in Figure 1a. First, an icosahedron is inscribed into a 2-sphere. The 12 vertices of this icosahedron yield the grid points (*ϕ*_*i*_, *θ*_*i*_) for the coarsest grid on *S*^2^ (black dots in Figure 1a). Each face of the icosahedron is an equilateral triangle. Further grid points are created at midpoints of the icosahedron edges and then scaled to lie on the 2-sphere (red dots in Figure 1a). The resulting grid has 42 grid points. The mid points of the icosahedron edges discretize each icosahedron face into four smaller equilateral triangles. The next finer grid is generated by creating mid points on their edges and scaling them to lie on the 2-sphere (blue dots in Figure 1a). The process can be iteratively repeated to obtain finer and finer grids. Obviously, this process directly generates only specific sets of grid points: 12, 42, 80, ... However, an arbitrary number of grid points can be obtained by creating the next largest grid and removing an appropriate number of points, a topic we also discussed in our previous publication.^28^*N*_*s*_ denotes the number of grid points on *S*^2^. By combining the *S*^2^ grid with the radial grid we obtain the translation grid with *N*_*r*_ × *N*_*s*_ grid points. Each grid point *i* is associated with a translation vector **t**_*i*_ = (*r*_*i*_, *ϕ*_*i*_, *θ*_*i*_). The grid points form *N*_*s*_ rays, each with *N*_*r*_ points, as depicted in Figure 2c).

We construct the rotation grid by systematically generating quaternions **q**_*j*_ using the polytope approach (polytope is an equivalent to polyhedron in higher-dimensional spaces). First, a 4-cube (tesseract) is inscribed in *S*^3^. A 4-cube is an four-dimensional analogue of a three-dimensional cube and is defined by 16 vertices . The prefactor 1/2 ensures that **q**_*j*_ is normalized to 1 and thus lies on *S*^3^. One can visualize a 4-cube as an object consisting of eight cubic cells (Figure 1b). The 16 vertices of the 4-cube yield the grid points **q**_*j*_ for the coarsest grid on *S*^3^ (black dots in Figure 1b). Further grid points are created by adding a point along each edge, face and center of the 4-cube, thereby subdividing each cubic cell into eight smaller cubes (red dots in Figure 1b). The new points are scaled to unit length to ensure that they lie on *S*^3^. As in Figure 1a, this process can be repeated iteratively to achieve finer and finer grids (e.g., blue dots in Figure 1b). In the last step, the orientation grid is truncated to the “upper half” of *S*^3^. *N*_*o*_ denotes the number of grid points on .

The full grid for the configuration space *SE*(3) is obtained as all possible combinations of translation and rotation grid points **x**_*ij*_ = (**t**_*i*_, **q**_*j*_). The total number of grid points is *N*_*d*_ = *N*_*r*_ · *N*_*s*_ · *N*_*o*_. Then the configurations of molecule *B*, , corresponding to grid points (**t**_*i*_, **q**_*j*_) are constructed in a two-step process. First, molecule *B* is placed in the reference configuration . That is, *B* is placed at the origin of the coordinate system and in a specific reference rotation . This reference rotation can have the axes of inertia aligned with the axes of the coordinate system, but this is not necessary. In the second step, the molecule *B* is first rotated by *R*(**q**_*j*_) and then translated by **t**_*i*_, where the transformation is applied to each atom *l* individually
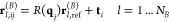
13The resulting configuration of molecule *B* can be represented in Cartesian coordinates  or in translational, rotational and internal coordinates .

### 2.4 Energy of Grid Cells

The energy associated with a grid cell (*ij*) is given by the effective potential . Obtaining *V*_eff_(**x**) requires a free-energy calculation^39^ for the six translational and rotational DoF and is computationally very costly. However, within the rigid-body approximation, the energy of the internal degrees of freedom is constant, and one can therefore replace the effective potential by the full (*N*_*A*_ + *N*_*B*_)-atom potential of the bimolecular system
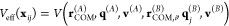
14

Thus, in principle, a single energy evaluation per grid point is sufficient. In practice, one might want to slightly improve this energy approximation using two strategies. First, to account for steric clashes, **v**_*A*_ and **v**_*B*_ can be relaxed while keeping the translational and rotational degrees of freedom of both molecules constrained. Second, to account for the fact that the energy is not entirely constant throughout the grid cell, *V*_eff_(**x**_*ij*_) can be calculated as a an average over a short simulation, where translational and rotational degrees of freedom of both molecules are restrained to remain close but not exactly equal to the set translation and orientation. Having obtained a valid expression of *V*_eff_(**x**_*ij*_) for each grid cell, the Boltzmann ratio in eq 7 can be evaluated according to eq 3.

### 2.5 Distances, Surfaces and Volumes of Grid Cells

The grid points **x**_*ij*_ induce a Voronoi-like tessellation^40^ of the six-dimensional translation and rotation space, where each point in this space is assigned to its closest grid point forming nonoverlapping grid cells. We defined these distances in terms of spherical coordinates (*r*, *θ*, *ϕ*) and in terms of angles between quaternions. The deviation from a Voronoi tessallation in Cartesian space are discussed in Section 4.4.

To calculate the distance between two adjacent grid points *h*_*αβ*_, the area of the intersecting surface of their grid cells  and the volume of a grid cell  in eq 7, we need to define a distance metric for the translation and orientation space. We will first discuss distance, surface and volume for translation and rotation space separately, before forming their product to discretize the SE(3) space. Throughout the discussion, we consider two adjacent grid points **x***_α_* = **x**_*ij*_ = (**t**_*i*_, **q**_*j*_) and **x**_*β*_ = **x**_*kl*_ = (**t**_*k*_, **q**_*l*_).

#### 2.5.1 Translation Grid

The translation grid is constructed from the radial and spherical subgrids. This leads to two types of adjacency relations: (i) radial neighbors (orange and blue cells in Figure 2d,e, and (ii) angular neighbors. Radial neighbors are stacked along one of the *N*_*s*_ radial rays in the translation grid. Their grid points have the same angular coordinates, but differ by one in the radial index: **t**_*i*_ = (*r*_*i*_, *ϕ*_*i*_, *θ*_*i*_) and **t**_*k*_ = *(r*_*k*=*i*±1_, *ϕ*_*k*=*i*_, *θ*_*k*=*i*_). Angular neighbors have the same radius but are neighbors on the spherical grid: **t**_i_*=* (*r_i_, ϕ_i_, θ_i_*) and **t**_*k*_ = (*r*_*k*=*i*_, *ϕ*_*k*_, *θ*_*k*_), where (*ϕ_i_*, *θ*_*i*_) ∼ (*ϕ*_*k*_, *θ*_*k*_).

The cells of the radial grid are separated by radii  at midpoints between grid points *r*_*i*_ as shown in Figure 2a. The distance metric on the radial grid is
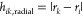
15The intersecting surface is calculated as the area of the *n*-sided spherical polygon with interior angles *α*_1_ . . . *α*_*N*_(41) (Figure 2b).
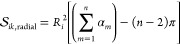
16

To define the Voronoi tesselation of the spherical grid,^42,43^ we use the angular distance
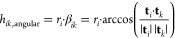
17where |**t**_*i*_| = r_*i*_ is the Euclidean length of the translation vector **t**_*i*_, and β_*ij*_ is the angle between **t**_*i*_ and **t**_*k*_. Within this distance metric, the points on a sphere that are closest to the coordinate pair (*ϕ*_*i*_, *θ*_*i*_) have the geometrical form of a spherical polygon (Figure 2b). The intersecting area is a part of the corresponding circular sector (Figure 2f) and can be calculated by subtracting the area of the circular sector with the smaller radius from the one with the larger radius
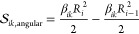
18where β_*ik*_ is given by eq 17.

In summary
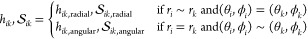
19

The volume of translation grid cells **r**_*i*_ is calculated from the corresponding sector of the sphere with radius *R*_*i*_, where sector volume is proportional to the surface of the grid cell:
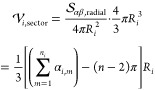
20The cell volume is obtained by subtracting the area of the next smaller sector  from 
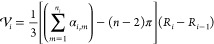
21where *R*_0_ = 0 and angles  are schematically shown in Figure 1b.

#### 2.5.2 Rotation Grid

The distance metric in rotation space^44^ is based on the angle between the unit quaternions **q**_*j*_ and **q**_*l*_ of two adjacent rotation grid cells
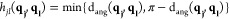
22with
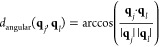
23Due to double coverage of the *S*^3^-hypersphere, the distance is defined as the minimum of the two values in eq 22.

The geometrical properties of 3-sphere Voronoi cells are difficult to picture directly, but an intuition can be built on analogy with the 2-sphere tessellation displayed in Figure 2b. In the S^2^ example, cells are spherical polygons and borders are spherical arcs between them, i.e., sections of S^1^. Intuition suggests that borders between cells in S^3^ could have the form of a section of S^2^, i.e., spherical polygons. We confirm this intuition by the following consideration.

Let *v*_1_...*v*_s_ be Cartesian coordinates of Voronoi vertices shared between neighboring hypersphere cells **q**_**j**_ and **q**_**l**_ (as a condition of neighborhood, cells must share at least three vertices). As they all share the property of equal distance to **q**_**j**_ and **q**_**l**_, they must lie on a hyperplane. However, as they are Voronoi vertices of hypersphere tessellation, they must also lie on a hypersphere. Thus, they lie on an intersection of a hyperplane and hypersphere, which can be an empty set, a point or a 2-sphere. The first two option imply that there is no intersecting hyper-surface between **q**_**j**_ and **q**_**l**_ and will not occur if **q**_**j**_ and **q**_**l**_ are adjacent. The third option tells us that the intersecting hyper-surface between **q**_**j**_ and **q**_**l**_ has the form of a sphere in three-dimensional space and we take advantage of this property to visualize and calculate its areas.

We devised the following algorithm to determine the area of the intersecting surface. If we stack the vertices *v*_1_...*v*_s_ that defined the intersecting hyper-surface, we obtain a 4 × *s* matrix **V**_4_. However, because we know they belong to a three-dimensional subspace (a sphere), there must exist a rotation rendering the fourth coordinate of all points equal zero. We find this rotation with singular value decomposition (SVD). Now, the rotated matrix can be interpreted as a 3× *s* matrix **V**_3_ and the vertices as points on a unit sphere that divide the spherical surface into two spherical polygons, the smaller of which is the border area we are looking for. This means that we can again use the formula given by eq 16 for *R*_*i*_ = 1 for analytical calculation of spherical polygon areas .

Finally, the volumes^1^ of hyperspherical Voronoi cells must be determined. To the best of our knowledge, there is no general analytic solution for this problem. To perform a numerical approximation, (higher-dimensional) triangulation can be performed by analogy of surface triangulation that is shown in Figure 2e. Additional 5000 points are selected at random on the hypersurface of a hypersphere and assigned to their corresponding Voronoi cells. On 2-spheres, Delaunay triangles^45^ are constructed from a dense set of points and their combined area approximates the area of a spherical section. Similarly, on 3-spheres, Delaunay triangulation leads to small tetrahedra filling a cell and their combined volumes are an approximation of a cell volume.

To confirm that the volumes of hypersphere Voronoi cells are reasonable, we compare them to the analytical value of unit hypersphere hyper-surface (what we call volume) π^2^ equally divided into 2*N*_*o*_ sections:
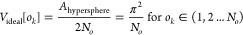
24

#### 2.5.3 Configurational Grid

To construct the configuration grid on *SE*(3), we combine translation and rotation grid. For two centers on the configuration grid, **x**_*α*_ = **x**_*ij*_ = (**t**_*i*_, **q**_*j*_) and **x**_*β*_ = **x**_*kl*_ = (**t**_*k*_, **q**_*l*_), to be adjacent they must share a point in one of the subgrids and must be adjacent in the other subgrid. That is
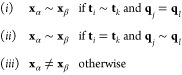
25The first case represents a transition in translation space, whereas the second case represents a transition in rotation space. The distance and surface between adjacent cells then are
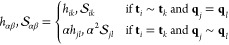
26where *h*_*ik*_ and  are given by eq 19, and *h*_*jl*_ and  are given by eqs 22 and 18. The grid cell volume is
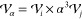
27where  is given by eq 21, and  is determined numerically.

In the combined space , the factor α represents the weight of the rotation space *SO*(3) relative to the translation space , and has been introduced in discussions of *SE*(3) robot manipulator spaces.^46^ We currently set α = 1.

*h*_*αβ*_,  and  can then be inserted into eq 7 to calculate *Q*_αβ,adjacent_.

### 2.6 Relation to Markov Models

The rate matrix **Q** and MSM transition matrix **T**(*τ*) are related by eq 6, and therefore share the same left and right eigenvectors^5^
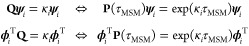
28where **ψ**_i_ are the right eigenvectors, and **ϕ**_i_ are the left eigenvectors, and λ_*i*_(*τ*_MSM_) =exp(κ_*i*_*τ*_MSM_) are the associated MSM eigenvalues. The definition of the rate matrix within the square-root approximation (eqs 5 and 7) enforces detailed balance
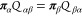
29Consequently, right and left eigenvectors are linked by diag(**π**)*ψ*_*i*_ = *ϕ*_i_, where **π** is the stationary distribution and is equal to the first left eigenvector.

**Figure 3:**
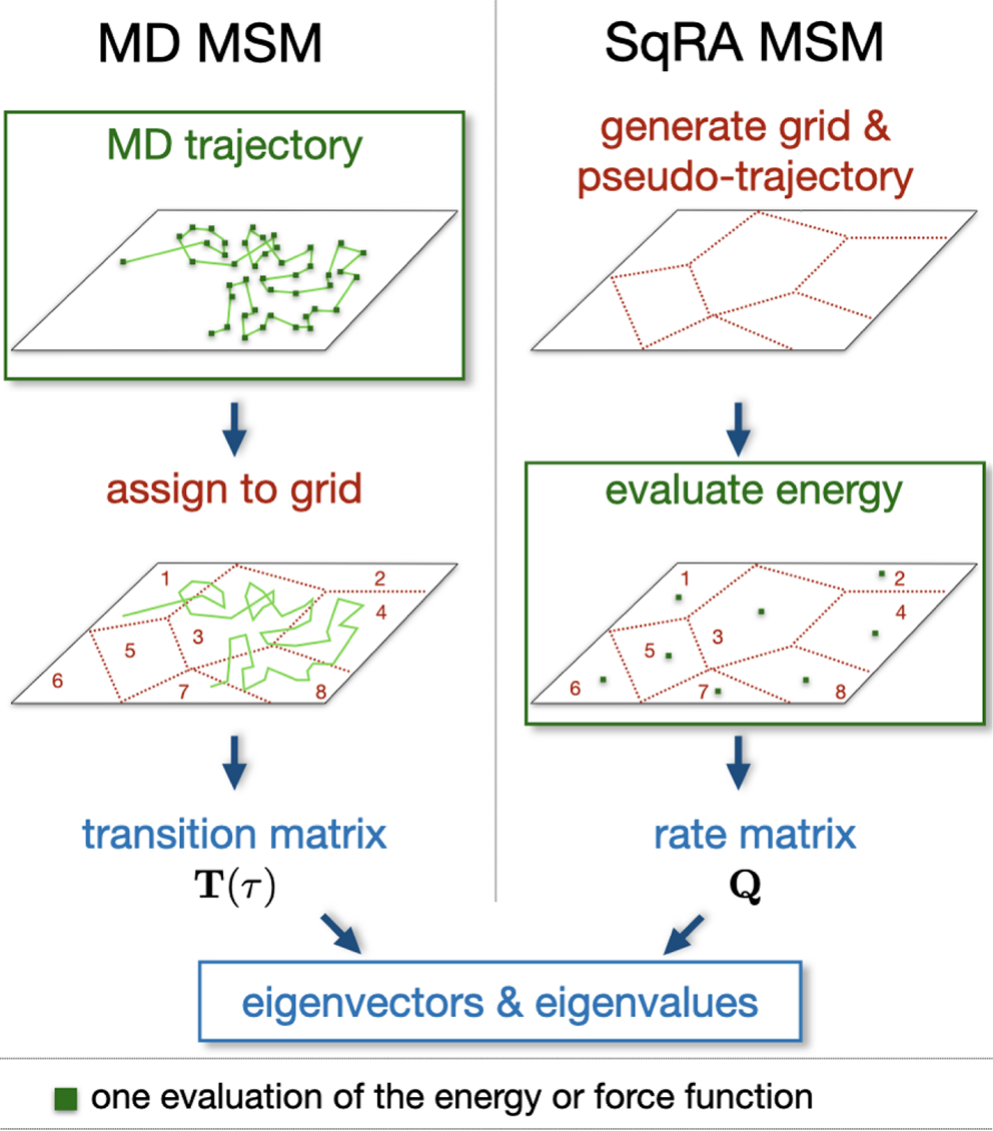
Comparison of the workflows for sampling-based and SqRA MSMs.

The dominant MSM eigenvectors contain a wealth of information on the metastable states and slow molecular processes. In a sampling-based MSM approach, they are obtained by estimating the elements of the MSM transition matrix *P*_*αβ*_(*τ*_MSM_) from an MD simulation. In the grid-based SqRA approach, they are obtained by evaluating the energy and geometric properties of each grid cell. Figure 3 compares the two approaches.

## 3 Computational Methods

### 3.1 Grid-Based Models

For the molecular association of two water molecules *A* and *B*, we constructed a grid based on *N*_r_ = 10 radial grid points, which were equally spaced between 0.2 and 0.4 nm, *N*_*s*_ = 80 angular grid points, and *N*_*o*_ = 80 rotational grid points. This yielded a grid with *N*_*d*_ = *N*_*o*_ × *N*_*s*_ × *N*_*r*_ = 64,000 grid cells. For the association of two C_60_F_2_ molecules, we used a radial grid with *N*_*r*_ = 10 points equally spaced between 0.8 and 1.3 nm, and the same angular and rotational grid as for the water dimer. For the association of bovine pancreatic trypsin inhibitor (BPTI) with trypsin, we used a radial grid with *N*_*r*_ = 10 points equally spaced between 3.5 and 4.5 nm, and the same angular and rotational grid as for the water dimer. Other settings for the radial grid are reported in the Supporting Information.

We calculated the volume  of each grid cell. We constructed the adjacency matrix of the grid and calculated the distances between adjacent cells *h*_*αβ*_ and the areas of the intersecting surfaces . Then, molecule *A* was placed at the origin of the coordinate system, and molecule *B* was translated and rotated to each of the grid cells as described in Section 2. The resulting 64,000 configurations were sequentially written to a .trr file (GROMACS trajectory format). The potential energy of each of these configurations was evaluated using GROMACS’s rerun command.The water molecules were modeled using the TIP3P water model.^47^ The C_60_F_2_ molecules were created in Avodagro^48^ by modifying the template for the C_60_ fullerene. The topology was created with the GROMOS-Topology-Builder.^49,50^ For the BPTI and Trypsine molecules the starting structure was PDB structure 4Y0Y^51^ with the two components separated and prepared as described in a previous publication of our group.^52^ From this information, we calculated the factor  = exp[(*V*_eff_(**x**_β_) – *V*_eff_(**x**_α_))/(2*k*_B_*T*)] for all pairs of adjacent cells. To avoid integer overflow, the energy difference *V*_eff_(**x**_β_) – *V*_eff_(**x**_α_) was capped at 500 kJ/mol. Energy differences of ≥500 kJ/mol correspond to transition rates that are numerically zero and can thus be safely set to zero. The (*N*_d_ × *N*_d_)-rate matrix **Q** was calculated using eq 7. The left and right eigenvectors and associated eigenvalues of the rate matrix were calculated with scipy’s eigenvalue solver.

The code we wrote to construct and evaluate grid-based models is formatted as a python 3.12.4^53^ package called molgri (for **mol**ecular **gri**ds) and the computational experiments formatted as snakemake 8.14.0^54^ pipelines for better reproducibility. Major Phython dependencies used in this project are numpy 1.26.4,^55^ scipy 1.13.1,^56^ networkx 3.3^57^ and mdanalysis 2.7.0.^58,59^

### 3.2 Spectral Clustering of **Q**

Spectral clustering was performed on the first six right eigenvectors of the rate matrix **Q**. The clustering algorithm used was KMeans^60^ as implemented in scikit-learn 1.5^61^ and the choice of 12 clusters was made.

### 3.3 Molecular Dynamics Simulations

Molecular dynamics simulations were conducted with GROMACS 2022^62−65^ and performed only for the system of two water molecules.

For the vacuum simulations, two water molecules were placed in a cubic box with 3 nm edge length. The interactions were modeled using the TIP3P water model.^47^ O–H bond lengths were constrained using LINCS algorithm.^66^ The dynamics were propagated using the built-in stochastic integrator for Langevin dynamics^67,68^ (setting sd) with a time step of Δ*t* = 2 fs. The reference temperature was set to 300 K, and we varied the coupling time in across different simulations runs: *τ*_c_ = 0.001 ps, 0.010 ps, 0.100 ps, 1.000 ps. Each simulation run was conducted for 4 × 10^7^ timesteps, corresponding to 80 ns simulation time. Long range interactions were cutoff at 1.4 nm. To prevent that the two molecules diffuse far beyond the maximum radius of the SqRA grid , we applied a flat-bottomed distance restraint (Figure 6) along the oxygen–oxygen distance *r*: no restraining potential for 0 ≤ *r* < 0.5 nm, harmonic potential with force constant *k* = 500 kJ/(mol nm^2^) for 0.5 nm ≤ *r* < 0.7 nm, and a linear restraining potential with force constant *k* = 500 kJ/(mol nm^2^) for *r* ≥ 0.7 nm. No pressure coupling was applied. Coordinates of the water molecules were written to a file every 5 timesteps.

For the simulations in explicit solvent, two water molecules were solvated with 2033 Lennard-Jones particles in cubic box with 3.6 nm edge length. The water molecules were modeled using the TIP3P water model.^47^ The Lennard-Jones particles had a mass of 4 atomic units, no charge and the following Lennard-Jones parameters: ϵ = 0.8202 kJ/mol and σ = 0.253 nm, approximately modeling a helium atom. The simulation parameters were the same as for the vacuum simulations with the exception of the long-range interactions, where we used Ewald summation with a long-range cutoff of 1.4 nm.

### 3.4 Markov State Models

From the MD simulation trajectories, we constructed Markov state models using the same grid as for the SqRA models. The trajectories were aligned to the reference translation and orientation of the first water molecule, and the translation and orientation of the second molecule was assigned to a grid cell using the following procedure: (1) the center-of-mass distance between the two molecules is calculated and the nearest cell center in the distance grid is selected, (2) the center-of-mass distance vector is scaled to unit length and the nearest cell center in the direction grid is selected, and (3) the rotation matrix between orientation of the reference structure and the orientation of molecule *B* is calculated and the nearest quaternion is selected from the rotation grid. The combination of the three assignments yields a grid cell index for each trajectory frame, and thus a microstate trajectory. The assignment is also implemented in our Python package molgri. To construct a Markov model from the microstate trajectory, we followed standard procedures.^5^ We constructed a MSM count matrix **C**(*τ*) by counting state-to-state transitions within lag time *τ*_MSM_. The resulting (*N*_s_ × *N*_s_)-matrix was stored in a sparse data format. We varied τ_MSM_ between 0.01 and 1.0 ps but always show *τ*_MSM_ = 0.1 ps in the results section. Detailed balance was enforced. The count matrix was row-normalized to obtain the MSM transition matrix **T**(*τ*). The left and right eigenvectors and associated eigenvalues were again calculated with scipy. Implied time scales were calculated as *t*_its,*i*_ = −*τ*_MSM_/ln(λ_*i*_(*τ*_MSM_)), where λ_*i*_(*τ*_MSM_) is the *i*th MSM eigenvalue.

## 4 Results and Discussion

### 4.1 SqRA Model of the Water Dimer

**Figure 4:**
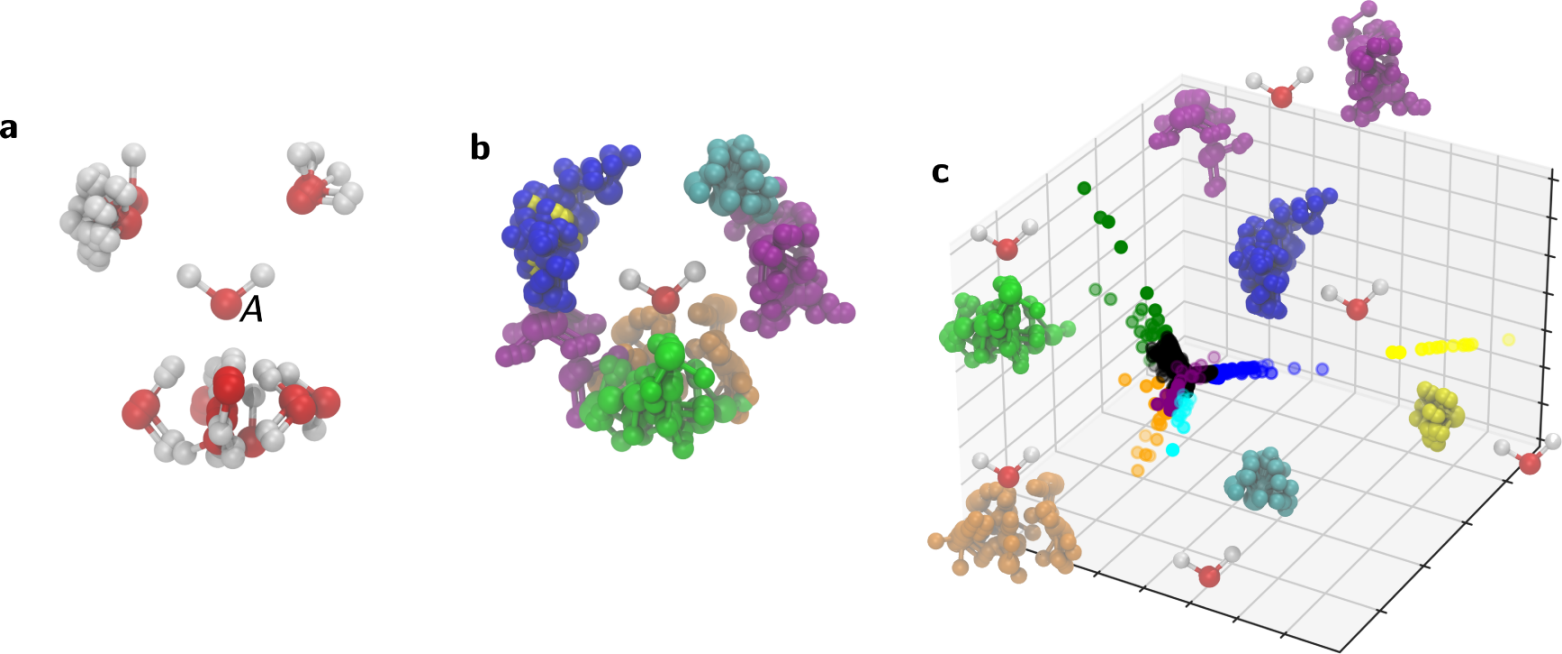
(a) Most probable configurations of the bimolecular system according to the stationary distribution calculated with the SqRA-Markov model. Molecule *A* is labeled, the rest of the structures represent molecule *B*. (b) K-Means clustering performed on the space of the first six eigenvectors of the rate matrix. Configurations that are part one cluster are shown in the same color. (The largest cluster with population 63834 and five tiny clusters with population <10 are not shown.) (c) A dot for each grid point is shown in the space of 1st, 2nd and 3rd eigenvector and colored according to the clustering in part b.

To illustrate the grid-based approach to molecular association, we consider two water molecules in vacuum and construct the SqRA-Markov model of the water dimer association on a configuration grid with 6.4 × 10^4^molgri grid cells. Figure 4a shows the highest-probability configurations of water molecule *B* relative to water molecule *A*, which were extracted from the stationary probability vector (first left eigenvector) of the SqRA rate matrix **Q**. The model correctly identifies configurations in which water *A* acts as a hydrogen-bond donor and configurations in which it acts as hydrogen-bond acceptor. Remarkably, in the hydrogen-bond-donor configurations, the rotation of water *B* is very restricted, whereas is its free to rotate in the hydrogen-bond-acceptor configuration. This free rotation, although at variance with the water-dimers of actual water molecules, is likely correct for the TIP3P water model, which does not account for the oxygen electron lone pairs. Further note that there is a difference in the number of left and right side hydrogen-bond donor configurations, which might be due to slight asymmetry in the discretization of the translation grid relative to the mirror plane of *A*.

To further analyze the dominant eigenspace of **Q**, we projected each grid center into the space of six dominant right eigenvectors and clustered in this six-dimensional space using the KMeans algorithm. Figure 4c shows that the eigenvectors of the rate matrix clearly separate the subspaces expected by chemical intuition: four clusters are found that correspond to the four possible hydrogen bonds with molecule *A* (shown in yellow, cyan, green and orange), where KMeans separates the large set of hydrogen-bond-acceptor configurations in two separate clusters (orange and green). The violet cluster represents the transition region between the two types of hydrogen bonding. There is an additional cluster (dark blue) that shows a broader region around one of the potential minima and five small clusters of 2–7 structures that seem to be artifacts of the choice of the number of clusters (not shown).

Also not shown is the most populated cluster containing over 99.7% of all generated poses which can be regarded as the set of all structures that have no particular importance to the slow processes of the system. This is a big contrast to the usual statistics of sampling-based methods, where a almost all sampled structures are found in the vicinity of the (few) deepest potential minima. The fact that a large majority of generated structures is not relevant to the binding of the two molecules might first seem like disappointing performance, but it is expected behavior for a grid that uniformly fills the configuration space. It is even desirable for two reasons: first, it allows us to identify transition states between low-energy configurations, such as the violet cluster; second, for a grid-based approach it is sufficient to reveal a single pose that lies inside a particular potential minimum, because the ensemble of structures within that minimum can be easily obtained in a subsequent step, either by applying a denser grid in the region of interest or by performing a short simulation starting from the identified structure.

The implied time scales of the SqRA-Markov model depend linearly on *τ*_c_ = ξ^–1^, ξ is the friction coefficient of the overdamped Langevin dynamics (eq 1). This friction coefficient is usually implemented as a thermostat coupling time *τ*_*c*_. By varying *τ*_*c*_ between 0.001 and 1.0 ps, the implied time scale of the slowest process decreases from 712 to 0.71 ps (see SI Figure S1). Since *τ*_*c*_ is an arbitrary parameter at this point, these values should not be assigned any chemical significance. Importantly, the eigenvectors remain largely unaffected by changes in the magnitude of the friction coefficient.

### 4.2 Comparison to MD

**Figure 5:**
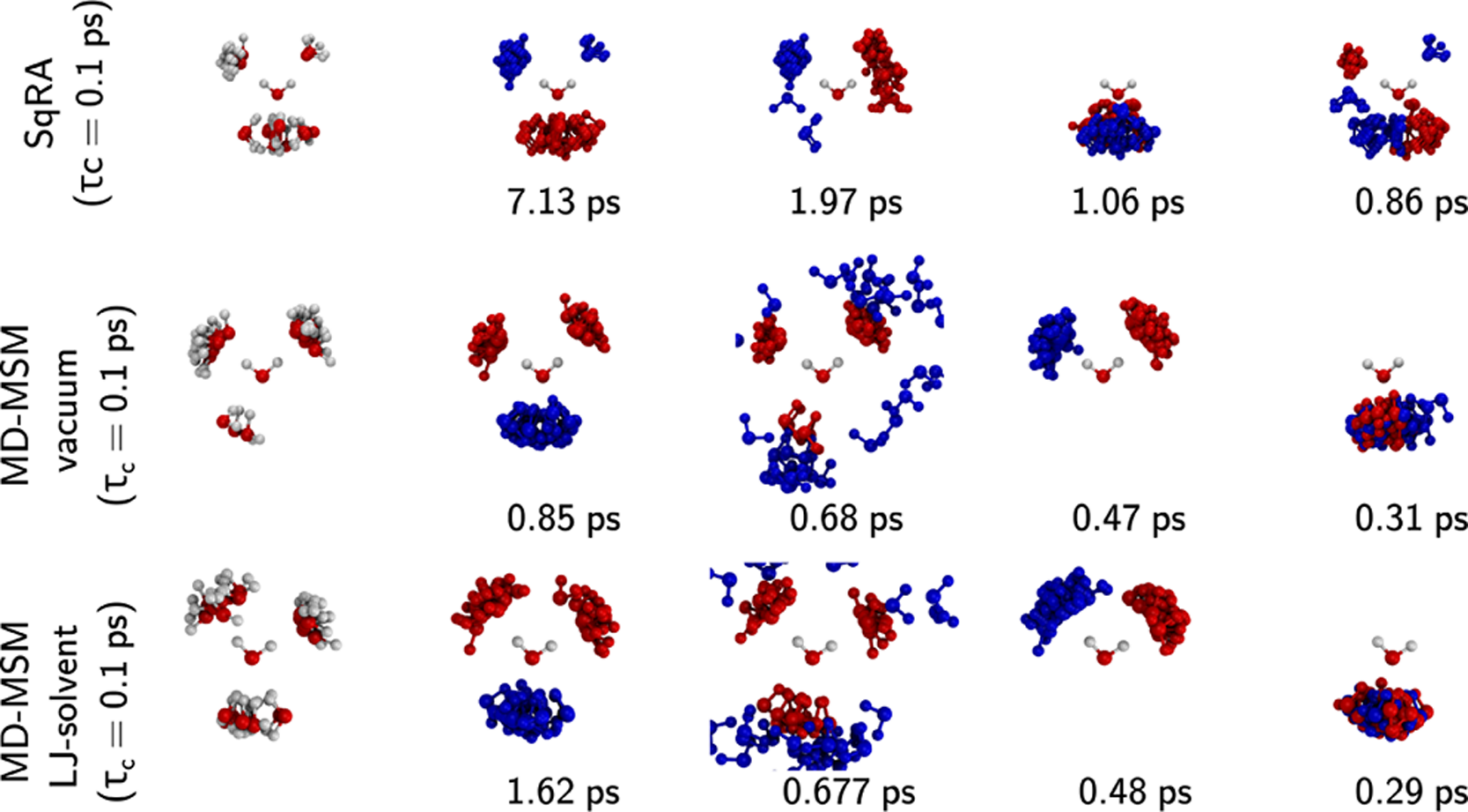
First five eigenvectors of water dimer system at three different simulation conditions. Top: SqRA model with *τ*_C_ = 0.1 ps, middle: 40 ns MD trajectory in vacuum with *τ*_C_ = 0.1 ps, bottom: 80 ns MD trajectory in a box of LJ particles with *τ*_C_ = 1 ps. In **blue**: structures corresponding to the 30 most negative values in the eigenvector; in **red**: structures corresponding to the 30 most positive values in the eigenvector. For the 0th eigenvector, structures corresponding to 30 eigenvector entries with the largest absolute value are shown. The implied time scales are noted below the corresponding eigenvector.

Figure 5 compares the SqRA-Markov model to MSMs built from MD simulations of two water molecules in vacuum. To prevent that differences in the discretization distort the results, we built the sampling-based MSMs on the grid with 6.4 × 10^4^ grid cells as the SqRA-Markov model. We sampled extensively (80 ns) to minimize the statistical error. Since the SqRA derivation assumes overdamped Langevin dynamics but typical molecular dynamics simulations are performed under underdamped conditions, we must enforce that the translation and rotation of the molecular system are in the overdamped regime. We try two simulation set-ups to fulfill this requirement: (i) simulating the bimolecular system in vacuum but with large friction constant or (ii) augmenting the thermostat noise with explicit solvent particles smaller than the solvate, in our case helium-like Lennard-Jones particles while setting *τ*_*c*_ to values conventionally used in MD. We expect the SqRA-MSM to align more closely with the vacuum simulation than with the Lennard-Jones solvent simulation. But relying on the thermostat as the main source of noise is a somewhat artificial setup, as friction and random forces in molecular systems naturally arise from the surrounding solvent. Thus, we include the second setup for comparison. However, the helium-like Lennard-Jones particles are comparatively large for the water dimer, so deviations from the ideal overdamped Langevin dynamics are expected.

The sampling-based MSM in vacuum identifies the same metastable states as the SqRA (first column in Figure 5), corresponding to two distinct states in which molecule *A* acts as a hydrogen-bond donor and one broad state in which molecule *A* acts as a hydrogen bond acceptor. In accordance with the SqRA-MSM, the sampling-based MSMs identify the exchange between hydrogen-bond acceptor state and hydrogen-bond donor states as a slow process (second column in Figure 5) and the exchange between the two hydrogen-bond donor states as a slightly faster process (fourth column in Figure 5).

As in the SqRA-Markov models, the eigenvectors are largely unaffected by the magnitude of *τ*_*c*_ (Figures S2–S5). In the vacuum simulations (Figures S2 and S3), the implied time scales decrease slightly with decreasing friction, but not orders of magnitude as in the SqRA-Markov model. In the simulations with explicit solvent (Figures S2 and S3), the implied time scales change minimally when *τ*_*c*_ is varied, because the friction predominantly arises from interactions with Lennard-Jones particles.

The sampling-based MSMs yield additional processes that are not part of the dominant eigenspace of the SqRA-Markov model. Specifically, we find processes that represent the exchange between inner and outer regions of the configuration grid (e.g., third eigenvector in row 2 in Figure 5). This prompted us investigate boundary conditions further, see the following section.

### 4.3 Boundary Conditions

**Figure 6:**
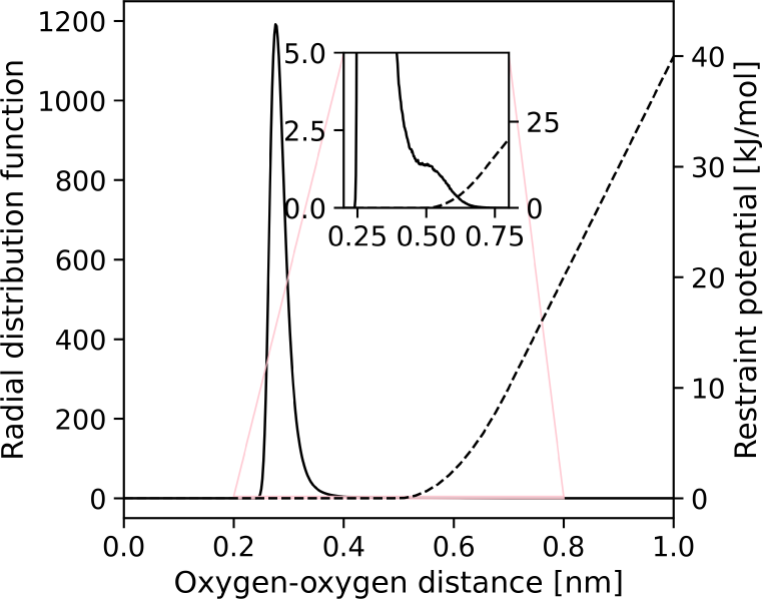
Radial distribution function (full line) and restraint potential (dashed line) for a MD run of two water molecules in a box of Lennard-Jones particles.

In our current SqRA model, transitions out of the grid into the bulk are not accounted for. A molecule in a boundary cell (one of the grid cells with the largest radial distance between molecules A and B) can diffuse to neighboring grid cells but its probability of diffusing through the outer surface between the current cell and the bulk is zero. Implementing these reflecting boundary conditions in an MD simulation is difficult, as a hard reflecting boundary or a strong restraining potential at the grid boundary will distort the dynamics in, at least, the outer grid cells. We therefore permitted unbinding and transitions across the grid boundary into the bulk. However, to prevent water molecule *B* from diffusing away from molecule *A*, we added a restraining potential to our system that starts increasing when oxygen–oxygen distance reaches 0.5 nm. This distance must be large enough to not disturb the bound structures of the system, which we confirmed in Figure 6 where the restraining potential is plotted alongside oxygen–oxygen radial distribution function from one of the MD simulations in explicit solvent. The radial distribution function of water in that figure might seem unusual, but the absence of second maximum and bulk limit is simply the consequence of simulating only two water molecules, where only the first solvation shell is present. Because of the restraining potential, we observe some would-be transitions into the bulk as bounces off the restraining potential. There is a small peak at around 0.5 nm in the distribution in Figure 6 that can be attributed to this bounce. To enforce the reflecting boundary conditions in our sampling-based MSMs, we considered two approaches to treat transitions out of the grid. In the first approach we include all distant out-of-grid configurations in the MSM estimation and assign them to the closest grid cell (closest orientation, closest direction and largest radius). This effectively extends the boundary cells of the translation grid indefinitely to *R* = ∞. In the second approach, distant out-of-grid configurations are assigned to none of the cells. When building the MS count matrix, transitions that either start or end with an unassigned structure are then omitted.

**Figure 7:**
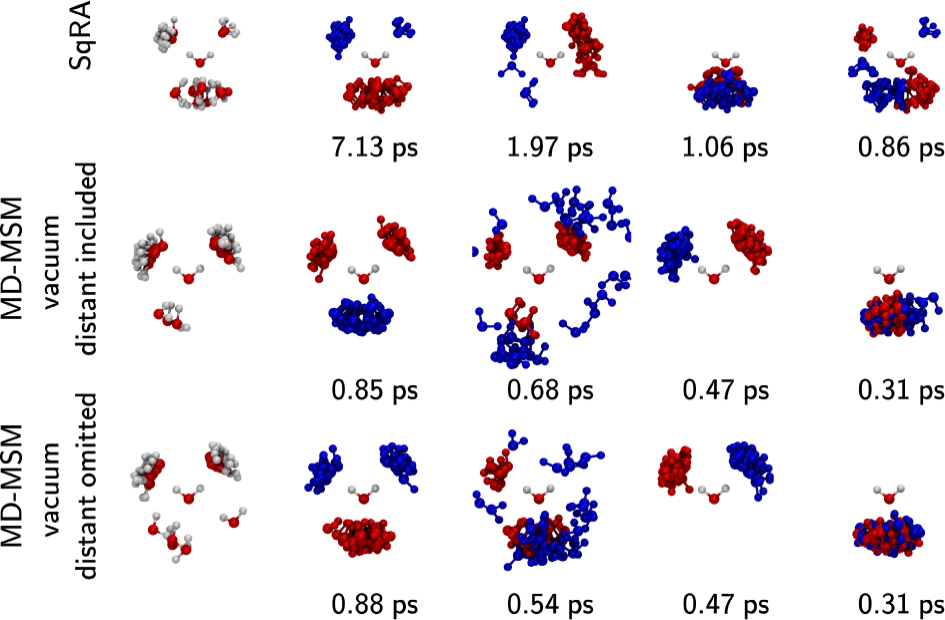
First five eigenvectors of the water dimer system at three different treatments of distant structures. Top: SqRA model, distant structures do not exist by construction, transition into this region is assumed impossible; middle: MD trajectory in vacuum, distant structures are assigned to the best available cell; bottom: MD trajectory in vacuum, distant structures are not assigned to any cell. Coupling time to a thermostat is *τ*_*C*_ = 0.1 ps in all three cases. For more information on distant structures, assigned and omitted approaches see text. For explanation of red/blue regions see description of Figure 5. The MD MSM in which distant structures were omitted featured a transition into a state with low population as a slow process. This is a known numerical artifact of MSMs and the eigenvector is therefore not shown.

Figure 7 illustrates the impact of the boundary treatment on sampling-based MSMs. The eigenvectors and eigenvalues of the two MSMs are nearly identical, except for the second eigenvector which represents the exchange between close and distant structures. As expected, including out-of-grid configurations in the MSM estimation causes this process to represent the exchange across the grid boundary into the bulk. When these configurations are omitted, this process still reflects a radial transition but is now confined within the grid. In the MD-MSM in which distant structures are omitted, the radial transitions appear to mix with transitions between metastable states in eigenvectors 4. It is important to note that the reflective boundary is highly artificial. We plan to extend the SqRA-Markov model to more accurately model transitions into the bulk, potentially by employing approaches such as those in ref (69).

### 4.4 Nonlinear Coordinates and Anisotropic Diffusion

**Figure 8:**
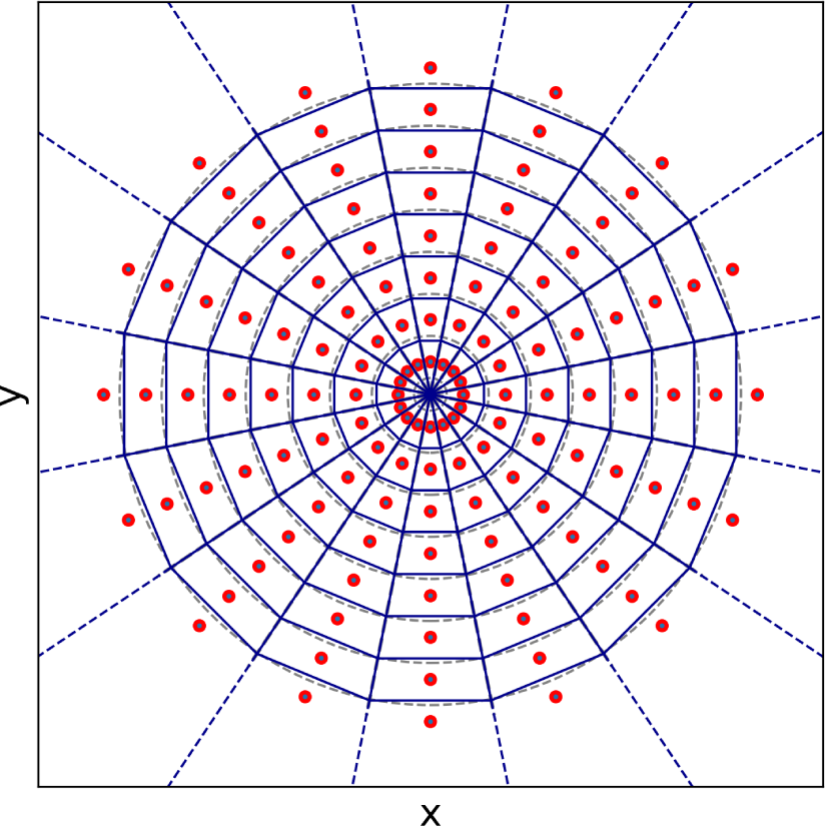
Comparison between Voronoi tessellation in polar coordinates (dashed gray lines) and Voronoi tessellation in Cartesian coordinates (blue grid) for a regular grid in polar coordinates (*r*, θ) (red dots).

We model translational diffusion in Cartesian coordinates in eq 2, but construct the translational grid in spherical coordinates. Specifically, we create the translational grid as a Voronoi tessellation in spherical coordinates and calculate the corresponding grid cell volumes, surfaces and distances accordingly. This approach induces a slight error in the prefactor  in eq 7. The derivation of this prefactor that the grid is a Voronoi tessellation in the same coordinate system as the Fokker–Planck operator, which, in this case, is the 3-dimensional Cartesian space. However, this deviation is likely minor because for a dense, regular spherical grid, the Voronoi tessellation in spherical coordinates closely resembles the equivalent in Cartesian coordinates, resulting in nearly identical grid cells. Figure 8 illustrates this effect in the 2-dimensional space for polar coordinates: red dots define a regular polar grid, and the Voronoi tessellation in polar coordinates (gray lines) almost perfectly overlaps with the Voronoi tessellation in the Cartesian coordinates (blue lines). For the translation grid in our model, it is however possible to replace the Voronoi tessellation in spherical coordinates by a Voronoi tessellation in Cartesian coordinates. We report the corresponding equations in the Supporting Information.

The situation is more complicated for the rotation grid, because rotational diffusion occurs in inherently nonlinear coordinates. This nonlinearity introduces an anisotropic diffusion tensor in the Fokker–Planck operator, meaning that *D* in eq 2 is no longer a constant but becomes a matrix . This matrix depends on the moments of inertia of the rotating molecule. The further the molecule’s shape deviates from spherical symmetry, the more **D** will deviated from *D*Id, where Id is the identity matrix. These deviations lead to inaccuracies in the current version of the SqRA-Markov model, which essentially assumes a spherical particle. A method for estimating the rotational diffusion tensor from MD simulations has been proposed in ref (70). To incorporate anisotropic diffusion into the SqRA-Markov model, we need an analytical expression for the rotational diffusion tensor and must rederive the prefactor in eq 7 for for anisotropic diffusion.

### 4.5 Computational Cost

Our grid-based approach scales to molecular systems that are considerably larger than a water dimer. Specifically, we tested a dimer of two fluorinated fullerenes C_60_F_2_–C_60_F_2_, and the protein–protein complex of bovine pancreatic trypsin inhibitor (BPTI) with trypsin.^52,71^ SqRA-MSM eigenvectors for these systems are reported in the Supporting Information in Figures SI 6 and SI 7, but should be interpreted with caution.

**Figure 9:**
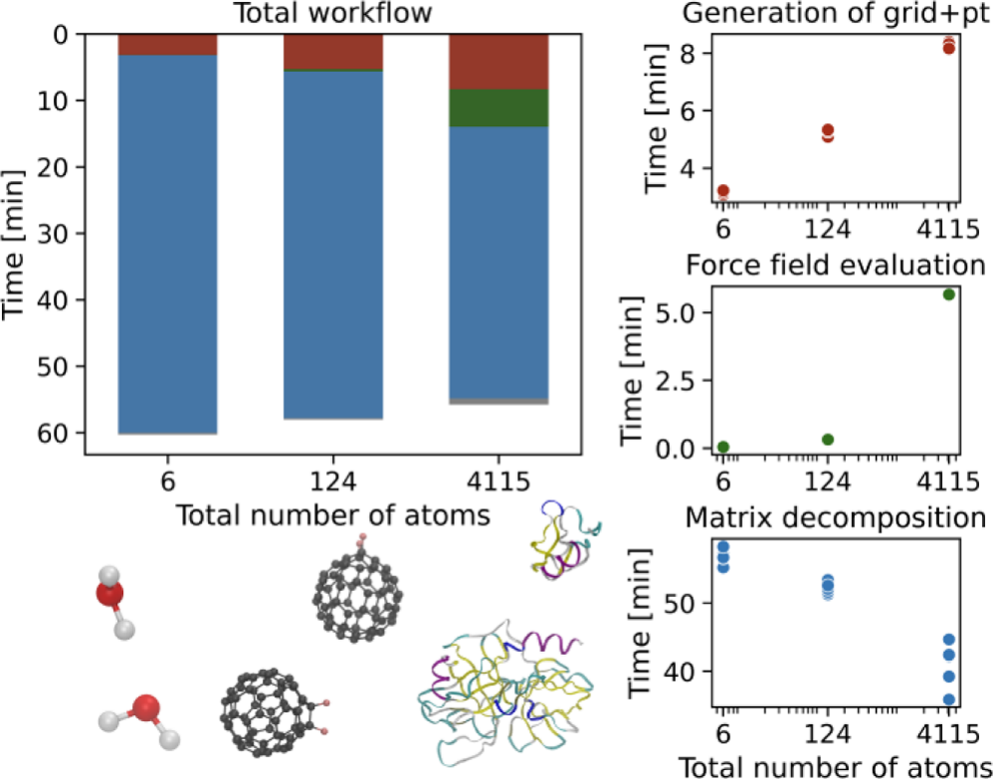
Comparison of time needed for the total molgri+SQRA workflow for systems spanning three different orders of magnitude in size: water dimer (6 atoms), fluorinated fullerene dimer (124 atoms) and Trypsine-BPTI system (4115 atoms). A 64,000-cell grid is used in all of the examples, only the radial distances are modified to account for different sizes of the molecules. The breakdown of the time needed for major steps of the process is shown on the right side. In addition to the separate steps, total time of the process includes some additional steps like file management and plot generation shown in gray on the total time plot. Runtime was measured five times for calculations running on up to ten cores of an Intel(R) Xeon(R) processor (2.20 GHz, 22 cores, 56 MB cache).

The computational cost of the grid-based models of our three test systems are shown in Figure 9, broken down by the workflow steps that were introduced in Figure 3. While all steps are orchestrated through the molgri software, only the first step is truly dependent on our algorithms, the energy calculation is handled by GROMACS^62−65^ and the decomposition is performed by the python package scipy.^56^ The total wall-clock time to calculate each of the three models on an Intel Xeon processor is about an hour, but the individual workflow steps scale differently with system size and merit a more detailed discussion.

The combined task of generating the grid and the pseudotrajectory increases in cost with the number of atoms *N*, but the scaling remains highly sublinear. The increase is caused by the generation of the pseudotrajectory. The sublinear scaling is expected, as transformations affect the center of mass and principal axes, and consequently the transformation does not need to be recomputed for each atom individually. Additionally, part of the system size effect arises from the increased cost of writing larger files as the number of atoms grows. The computational cost of generating the underlying grid is entirely independent of the number of atoms *N* and only depends on the number of grid points *N*_*d*_. In general, the cost of generating the grid scales superlinearly with the number of grid points. However, we implemented an efficient network-based representation of polygons, ensuring that grid generation remains a matter of seconds. The combined cost for grid and pseudotrajectory generation for *N*_*d*_ = 64,000 is thus less than 10 min.

The computational cost of the energy evaluation rapidly increases with system size, where the exact scaling depends on the type of molecular energy function and how this function is implemented. Empirical force fields, as used in this study, typically scale as *N* ln *N* and can be calculated so efficiently that the 64’000 energy evaluations for each system are completed within seconds for the water and the fullerene system, and within slightly more than 5 min for the protein-dimer. Thus, despite the steep scaling, energy evaluation constitutes the smallest contribution to the overall computational cost in our test systems. However, the grid-based approach is not restricted to empirical force fields. More expensive energy functions, such as energies based on quantum chemical calculations, can be used in lieu of a force field.

In our current setup, the largest contribution to the computational cost is the eigendecomposition of the rate matrix. The time needed and the success of eigendecomposition of a large sparse matrix **Q** depends strongly on how well-conditioned the matrix is. Therefore, we observe changes in speed that are not directly related to the system size *N* or the grid-size *N*_*d*_. The decreasing trend in Figure 9 is probably coincidental. Another critical resource in matrix decompositions is the computer memory, which currently limits our approach to  grid points.^25^

### 4.6 Comparison with Other Grid-Based Methods

Although our method is not primarily designed for protein–ligand binding, it is informative to compare it to molecular docking approaches^72−74^ and sampling-based methods for protein–ligand binding, such as MSMs^7−9^ and SEEKR2.^75,76^

The objective of molecular docking is to generate potential configurations of a protein–ligand system, often using grids in translational and rotational space, and to identify low-energy configurations through a single energy calculation per grid point. In these two aspects, our method resembles molecular docking. Specifically, because SqRA-MSMs also require only a single energy calculation per grid point, our grid-based approach is almost as computationally efficient as docking, potentially enabling high-throughput screening of binding partners.

Molecular docking cannot provide kinetic information because, to accurately calculate the probability flux between neighboring grid cells, the grid must meet specific requirements. First, it needs to be a Voronoi grid. Second, the grid cells should be small and should be ideally of equal size. Third, the geometric parameters of the grid cell must be known. Our work has therefore focused on generating uniform Voronoi grids^28^ and on deriving analytical expressions for their geometric parameters (Section 2). With these two requirements in place, one can construct SqRA-MSMs for molecular association, which give access to metastable states and competing binding pathways, along with the associated time scales. Since SqRA-MSMs require small grid cells, we generate around  configurations for a molecular association process, approximately 10 to 100 times more than a typical docking run.

Sampling-based approaches for modeling molecular association also frequently use grids to describe the kinetics, estimating the probability flux between neighboring cells by monitoring the cell-to-cell transitions in simulations. This approach has two major drawbacks compared to the SqRA-MSMs. First, accurately estimating the probability flux requires multiple crossings of cell boundaries, demanding thousands of simulation time steps and force evaluations–far more computationally intensive than the single force evaluation per grid cell in SqRA-MSMs. Second, it is challenging to ensure convergence, as these simulations must thoroughly sample both translational and rotational space. Additionally, the rates and metastable states in sampling-based MSMs are sensitive to statistical noise in flux estimates, making accuracy difficult to achieve and control. By contrast, the accuracy of a SqRA-MSM can be fully controlled by adjusting the space covered by the grid and the grid resolution.

A major advantage of sampling-based approaches compared to SqRA-MSMs is that simulations do not (usually) rely on the rigid body assumption. Therefore, sampling-based approaches naturally account for conformational flexibility of the two molecules and for solvent effects, which we know to be major contributors to molecular association. We anticipate that these effects could be incorporated into our method by including explicit solvent molecules to the energy calculation and by performing a short energy minimization while restraining the system to the grid cell. An alternative approach would be to average the results over a brief MD simulation. Achieving fully accurate energy values for each grid cell would ideally require constructing a free-energy surface,^77^ although this is computationally expensive in six dimensions. In SEEKR2,^75,76^ each individual grid cell can use a distinct simulation technique. This approach allows data from multiple sources, each with different computational demands, to be seamlessly integrated into a unified multiscale kinetic model. A similar strategy could potentially be applied to link SqRA-MSM with sampling-based approaches.

Overall, our grid-based approach to molecular association, in its current version, is best suited for molecular systems which require a computationally demanding energy function and are well approximated by assuming rigid-body behavior.

## 5 Conclusion and Outlook

The grid-based approach to molecular association offers significant computational advantages, as it requires only a single energy evaluation per grid cell, making it highly efficient. Notably, the number of grid points does not increase with system size, allowing the method to be applied to large molecular systems. With  to  grid points, this approach is compatible with computationally expensive energy functions, including energies based on electronic structure calculations.

We implemented the grid-based approach to molecular association in the python package MolGri. In its current version, the MolGri package offers a systematic approach to generating configurations for molecular association processes and analyzing their energies. This functionality makes it immediately valuable for studies of molecular association and for producing input structures for electronic structure calculations. MolGri can also be used to generate cluster or solvation shell configurations by first constructing a dimer grid, extracting the low-energy configurations, and then iteratively adding more molecules, using the extracted configurations as molecule *A* in subsequent steps.

MolGri can also be employed to construct SqRA-Markov models, which we have demonstrated to accurately identify the metastable states of molecular association processes. These models provide insight into the long-range interactions that steer molecular association and the underlying binding mechanism. However, the current implementation does not yet yield dynamically accurate results. The primary limitations are the neglect of transitions into the bulk and the omission of anisotropic rotational diffusion, which we aim to address in the next version of the model.

In summary, this grid-based method significantly reduces the number of energy evaluations required compared to MD simulations of molecular associations processes, while still offering a comprehensive view of the configuration space and estimates of key transition kinetics. Its potential applications span a range of fields, including dimer formation, nanoparticle growth, molecular self-assembly, protein–ligand binding, host–guest systems, and chemical reactions.

## Data Availability

The python package MolGri can be installed from PyPi (pip install molgri) or from the development repository on GitHub: (https://github.com/bkellerlab/molecularRotationalGrids).

## Supporting Information Available

Square-root approximation, water models, larger systems, Voronoi tessellation in Cartesian coordinates for the translational grid, and difference between Voronoi grids in spherical and Cartesian coordinates (PDF)

## Footnotes

1 The difference between the mathematical objects *sphere* and *ball* is important here. We are interested in the three-dimensional object (embedded in 4D) of a 3-sphere, which is the surface of the 3-ball. The volumes of our hypersphere cells are sections of the 3-sphere. We are at no point interested in the 4D volume of a 3-ball.
