# Supplementary material for: Toward Grid-Based Models for Molecular Association
Source: J Chem Theory Comput. 2025 Jan 13;21(2):614–28. doi: 10.1021/acs.jctc.4c01293 (PMC11780749; doi:10.1021/acs.jctc.4c01293)
Supplement: Supplementary file 1 — ct4c01293_si_001.pdf [file ct4c01293_si_001.pdf]

SI: Towards grid-based models for molecular association

Hana Zupan and Bettina G. Keller

(Dated: 27 November 2024)

Keywords: Suggested keywords

I. SQUARE-ROOT APPROXIMATION

A. Overdamped Langevin dynamics

Consider a molecular system with N atoms and $3N$ positional degrees of freedom. A collective variable x_i is a function that maps the $3N$ positional degrees of freedom to a real number: $x_i : \mathbb{R}^{3N} \mapsto \mathbb{R}$. We assume that in a low dimensional collective variable space $\mathbf{x} = (x_1, x_2 \dots x_m) \in \Omega \subset \mathbb{R}^m$ the dynamics of the system can be modelled by overdamped Langevin dynamics [1]:

$$\mathrm{d}\mathbf{x}(t) = \boldsymbol{\mu}(\mathbf{x}(t))dt + \sigma \mathrm{d}\mathbf{B}(t)\,, \tag{1}$$

where $\mathbf{B}(t) = (B_1(t) \dots B_m(t))$ is an m-dimensional Wiener process, $\boldsymbol{\mu}$ is the m-dimensional drift vector

$$\boldsymbol{\mu}(\mathbf{x}(t)) = -\xi^{-1} M^{-1} \nabla V_{\text{eff}}(\mathbf{x}(t))\,, \tag{2}$$

and

$$\sigma = \sqrt{2 k_B T \xi^{-1} M^{-1}}\,, \tag{3}$$

scales the Wiener process and is linked to the diffusion of the system in the collective variables space. We assume that the diffusion is isotropic in the collective variable, and hence σ is is simply a scalar. For non-isotropic diffusion, σ has to be replaced by an $(m \times m)$-matrix. In eq. 2 and 3, ξ is a friction parameter with units s^{-1}, M is a the effective mass, $V_{\text{eff}} : \Omega \mapsto \mathbb{R}$ is the effective potential for the collective variable space, k_B is the Boltzmann constant, T is the temperature, and $\nabla f(\mathbf{x}) = (\partial f/\partial x_1, \dots \partial f/\partial x_m)^\top$ denotes the gradient of a function $f : \mathbb{R}^m \to \mathbb{R}$.

B. Fokker-Planck equation

$\rho(\mathbf{x}, t)$ is a probability density on the space of collective variables, whose time-evolution is governed by the Fokker-Planck equation. The Fokker-Planck equation associated to overdamped Langevin dynamics (eq. 1) is [1]

$$\begin{aligned}\frac{\partial}{\partial t}\rho(\mathbf{x},t) &= -\sum_{i=1}^{m} \frac{\partial}{\partial x_i}\left[\mu_i(\mathbf{x},t)\cdot\rho(\mathbf{x},t)\right] + D\sum_{i=1}^{m}\frac{\partial^2}{\partial x_i^2}\rho(\mathbf{x},t) \\ &= -\nabla\cdot\left[\boldsymbol{\mu}(\mathbf{x}(t))\cdot\rho(\mathbf{x},t)\right] + D\nabla\cdot\nabla\rho(\mathbf{x},t) \\ &= \mathcal{Q}\rho(\mathbf{x},t)\,. \end{aligned} \tag{4}$$

where $\mathcal{Q}$ is the Fokker-Planck operator. For a vector field $\mathbf{f}(\mathbf{x}) = (f_1(\mathbf{x}), \dots f_m(\mathbf{x})) \in \mathbb{R}^m$, $\nabla \cdot \mathbf{f}(\mathbf{x}) = (\partial/\partial x_1 \dots \partial/\partial x_m) \cdot (f_1(\mathbf{x}), \dots f_m(\mathbf{x})) = \partial/\partial x_1 f_1(\mathbf{x}) + \dots \partial/\partial x_m f_m(\mathbf{x})$ denotes the divergence of the vector field [2]. $D = \sigma^2/2 = k_B T \xi^{-1} M^{-1}$ is the diffusion constant.

The stationary density associated to eq. 4 is the Boltzmann density

$$\pi(\mathbf{x}) = \frac{\exp\left(-\frac{1}{k_B T} V_{\text{eff}}(\mathbf{x})\right)}{Z} \tag{5}$$

where $Z = \int_\Omega \mathrm{d}\mathbf{x}\, \exp\left(-\frac{1}{k_B T} V_{\mathrm{eff}}(\mathbf{x})\right)$ is the configurational partition function. For this density, $\frac{\partial}{\partial t}\pi(\mathbf{x}) = \mathcal{Q}\pi(\mathbf{x}) = 0$.

C. Voronoid grid and Master equation

The collective variable space is discretized into N_d non-overlapping Voronoi cells $\Omega_1, \dots \Omega_{N_d}$. The center of the Voronoi cell Ω_α is denoted $\mathbf{x}_\alpha$. Its cell volume is

$$\mathcal{V}_\alpha = \int_{\Omega_\alpha} \mathrm{d}\mathbf{x}\, 1\,. \tag{6}$$

The surface $\mathcal{S}_\alpha$ of a Voronoi cell Ω_α consists of the intersecting surfaces $\delta\Omega_\alpha \delta\Omega_\beta$ between Ω_α and its adjacent cells Ω_β. These surface areas are $(m-1)$-dimensional hyper-planes, and their surface areas are defined as

$$\mathcal{S}_{\alpha\beta} = \oint_{\delta\Omega_\alpha\delta\Omega_\beta} \mathrm{d}S(\mathbf{z})\, 1\,. \tag{7}$$

The total surface area of Ω_α then is

$$\mathcal{S}_\alpha = \sum_{\beta\sim\alpha} \mathcal{S}_{\alpha\beta}\,, \tag{8}$$

where $\beta \sim \alpha$ indicates all Ω_β adjacent to Ω_α.

When represented on this grid, the time-dependent probability density $\rho(\mathbf{x}, t)$ becomes a time-dependent vector

$$\boldsymbol{\rho}(t) : \rho_\alpha(t) = \int_{\Omega_\alpha} \mathrm{d}\mathbf{x}\, \rho(\mathbf{x}, t) \tag{9}$$

and evolves according to a master equation [3, 4].

$$\begin{aligned} \frac{\mathrm{d}}{\mathrm{d}t}\rho_\beta(t) &= \sum_{\alpha\neq\beta} Q_{\alpha\to\beta}\, \rho_\alpha - \sum_{\alpha\neq\beta} Q_{\beta\to\alpha}\rho_\beta \\ &= \sum_{\alpha=1}^{N_d} Q_{\alpha\beta}\, \rho_\alpha\,, \end{aligned} \tag{10}$$

where $Q_{\alpha\to\beta}$ represents the transition rate constant from Ω_α to Ω_β. The first sum in eq. 10 represents the density that flows into Ω_β from any other grid cell Ω_α, and the second sum represents the density that flows out of Ω_β into any other grid cell Ω_α. In the second line, we reformulated the master equation as matrix-vector equation by setting $Q_{\alpha\to\beta} = Q_{\alpha\beta}$, $Q_{\beta\to\alpha} = Q_{\beta\alpha}$, and $Q_{\alpha\alpha} = -\sum_{\alpha\neq\beta} Q_{\beta\alpha}\rho_\beta$.

$$\frac{\mathrm{d}}{\mathrm{d}t}\boldsymbol{\rho}^\top(t) = \boldsymbol{\rho}^\top(t)\mathbf{Q} \tag{11}$$

where $\boldsymbol{\rho}^\top$ denotes the transpose of $\boldsymbol{\rho}$. Eq. 11 is the discretized version of the Fokker-Planck equation (eq. 4), and the rate matrix $\mathbf{Q}$ is the discretized Fokker-Planck operator $\mathcal{Q}$.

To be consistent with the convention in Markov state models [5], we chose a convention in which the row-sum of the rate matrix is zero $\sum_{\beta=1}^{N_d} Q_{\alpha\beta} = 0$. Frequently [4], the rate matrix elements are defined as $Q_{\alpha\to\beta} = \widetilde{Q}_{\beta\alpha}$, where the resulting rate matrix $\widetilde{\mathbf{Q}}$ is the transpose of $\mathbf{Q}$. In this case, the time evolution of the density vector is obtained by multiplying it from the right to $\widetilde{\mathbf{Q}}$.

We additionally assume that in the limit of infinitesimally small time intervals $\mathrm{d}t$, probability density is only transferred between directly neighboring cells, such that $Q_{\alpha\beta} = 0$ if Ω_α and Ω_β are not adjacent. Overall, we obtain the following structure for the rate matrix

$$Q_{\alpha\beta} = \begin{cases} Q_{\alpha\beta,\,\mathrm{adjacent}} & \alpha\sim\beta \\ 0 & \alpha\not\sim\beta \\ -\sum_{\beta=1,\beta\neq\alpha}^{N_d} Q_{\alpha\beta,\,\mathrm{adjacent}} & \alpha=\beta\,. \end{cases} \tag{12}$$

D. Square-root approximation of the Fokker-Planck operator

The goal of the Square-root approximation of the Fokker-Planck operator [6–9] is to find an analytical expression for the rate constants between adjacent cells. Using Gauss's divergence theorem [10], one can show that [6–8]

$$\begin{aligned} Q_{\alpha\beta,\mathrm{adjacent}} &= \frac{1}{\pi_\alpha} \oint_{\delta\Omega_\alpha\delta\Omega_\beta} \mathrm{d}S(\mathbf{z})\, \Phi(\mathbf{z})\pi(\mathbf{z}) \\ &= \frac{1}{\pi(\mathbf{x}_\alpha)\mathcal{V}_\alpha} \Phi_{\alpha\beta} \oint_{\delta\Omega_\alpha\delta\Omega_\beta} \mathrm{d}S(\mathbf{z})\, \pi(\mathbf{z})\,, \end{aligned} \tag{13}$$

where $\mathbf{z} \in \Omega$ is a point in the collective variable space and $\oint_{\delta\Omega_\alpha\delta\Omega_\beta} \mathrm{d}S(\mathbf{z})$ is a surface integral over the intersecting surface $\delta\Omega_\alpha\delta\Omega_\beta$ between Ω_α and Ω_β. $\Phi(\mathbf{z})$ is a flux factor and on the second line, we assumed that $\Phi(\mathbf{z})$ is a constant over the intersecting surface and can thus be replaced by a constant $\Phi_{\alpha\beta}$. π_α represents the stationary probability to find the system in grid cell Ω_α. In the second line, we additionally assume that the effective potential energy is approximately constant within Ω_α and can therefore be represented by the effective potential energy at the cell center

$$V_{\mathrm{eff}}(\mathbf{x})|_{\mathbf{x}\in\Omega_\alpha} \approx V_{\mathrm{eff}}(\mathbf{x}_\alpha)\,. \tag{14}$$

Then $\pi(\mathbf{x})|_{\mathbf{x}\in\Omega_\alpha} \approx \pi(\mathbf{x}_\alpha)$, and π_α is given as

$$\pi_\alpha = \int_{\Omega_\alpha} \mathrm{d}\mathbf{x}\, \pi(\mathbf{x}) = \pi(\mathbf{x}_\alpha)\mathcal{V}_\alpha\,. \tag{15}$$

Similarly, the time-dependent probability density vector is

$$\boldsymbol{\rho}(t) : \rho_\alpha(t) = \int_{\Omega_\alpha} \mathrm{d}\mathbf{x}\, \rho(\mathbf{x},t) = \rho(\mathbf{x}_\alpha,t)\mathcal{V}_\alpha\,. \tag{16}$$

To approximate the integral in eq. 13, we approximate the effective potential on the intersecting surface as the arithmetic mean of the effective potential at the two cell centers

$$V_{\mathrm{eff}}(\mathbf{x})|_{\mathbf{x}\in\delta\Omega_\alpha\delta\Omega_\beta} \approx \frac{V_{\mathrm{eff}}(\mathbf{x}_\alpha) + V_{\mathrm{eff}}(\mathbf{x}_\beta)}{2} \tag{17}$$

The stationary density on the intersecting surface then is

$$\pi(\mathbf{x})|_{\mathbf{x}\in\delta\Omega_\alpha\delta\Omega_\beta} \approx \frac{1}{Z}\exp\left(\frac{1}{k_BT}\frac{V_{\mathrm{eff}}(\mathbf{x}_\alpha) + V_{\mathrm{eff}}(\mathbf{x}_\beta)}{2}\right) = \sqrt{\pi(\mathbf{x}_\alpha)\pi(\mathbf{x}_\beta)} \tag{18}$$

Eq. 18 is the square-root approximation. With this approximation, the transition rate constant between adjacent cells is

$$Q_{\alpha\beta,\mathrm{adjacent}} = \frac{1}{\pi_\alpha}\Phi_{\alpha\beta}\sqrt{\pi(\mathbf{x}_\alpha)\pi(\mathbf{x}_\beta)} \oint_{\delta\Omega_\alpha\delta\Omega_\beta} \mathrm{d}S(\mathbf{z})\, 1 = \Phi_{\alpha\beta}\frac{\mathcal{S}_{\alpha\beta}}{\mathcal{V}_\alpha}\sqrt{\frac{\pi(\mathbf{x}_\beta)}{\pi(\mathbf{x}_\alpha)}}\,, \tag{19}$$

E. Flux factor

The assumption that the flux factor $\Phi(\mathbf{x})$ does not depend on the position implies that it does not depend on the effective potential $V_{\mathrm{eff}}(\mathbf{x})$. We therefore can derive an expression for Φ from the Fokker-Planck equation at constant potential

$$\frac{\partial}{\partial t}\rho(\mathbf{x},t) = D\nabla\cdot\nabla\rho(\mathbf{x},t)\,, \tag{20}$$

that is, by setting the drift term in eq. 4 to zero. Eq. 20 is Fick's second law of diffusion [11]. At constant potential the square-root approximation of the transition rate constant (eq. 19) is

$$Q_{\alpha\beta,\text{adjacent}} = \Phi_{\alpha\beta}\frac{\mathcal{S}_{\alpha\beta}}{\mathcal{V}_\alpha}\,, \tag{21}$$

because $\pi(\mathbf{x}_\beta) = \pi(\mathbf{x}_\alpha)$.

To derive an expression for the flux factor [9], we integrate both sides of eq. 20 over Ω_β and obtain an evolution equation for the probability $\rho_\beta(t)$ in grid cell Ω_β

$$\frac{\mathrm{d}}{\mathrm{d}t}\rho_\beta(t) = \int_{\Omega_\beta}\mathrm{d}\mathbf{x}\frac{\partial}{\partial t}\rho(\mathbf{x},t) = \int_{\Omega_\beta}\mathrm{d}\mathbf{x}\; D\nabla\cdot\nabla\rho(\mathbf{x},t)\,, \tag{22}$$

The right-hand side is calculated using Gauss's divergence theorem

$$\begin{aligned} D\int_{\Omega_\beta}\mathrm{d}\mathbf{x}\;\nabla\cdot\nabla\rho(\mathbf{x},t) &= D\oint_{\delta\Omega_\beta}\mathrm{d}S(\mathbf{z})\,(\nabla\rho(\mathbf{x},t))\cdot\mathbf{n}(\mathbf{z}) \\ &= D\sum_{\alpha\sim\beta}\left(\nabla\rho(\mathbf{x},t)|_{\mathbf{x}=\mathbf{x}_\beta}\right)\cdot\mathbf{n}_{\beta\alpha}\mathcal{S}_{\beta\alpha}\,, \end{aligned} \tag{23}$$

where $\oint_{\delta\Omega_\beta}\mathrm{d}S(\mathbf{z})$ is a surface integral over the hull of Ω_β, and $\mathbf{n}(\mathbf{z})$ is the unit normal vector on this surface at position $\mathbf{z}$. In the second line we used that Ω_β is a Voronoi cell, and therefore the surface integral is equal to summing over the intersecting surfaces $\delta\Omega_\beta\delta\Omega_\alpha$ between Ω_β and its adjacent cells Ω_α.

$$\mathbf{n}_{\beta\alpha} = \frac{\mathbf{x}_\alpha - \mathbf{x}_\beta}{|\mathbf{x}_\alpha - \mathbf{x}_\beta|} \tag{24}$$

is the unit normal vector on this intersecting surface, where $|\mathbf{x}_\alpha - \mathbf{x}_\beta|$ is the Euclidean norm.

The term $(\nabla\rho)\cdot\mathbf{n}_{\beta\alpha}$ in eq. 23 is a directional derivative [12] along $\mathbf{n}_{\beta\alpha}$ and can be approximated as

$$\left(\nabla\rho(\mathbf{x},t)|_{\mathbf{x}=\mathbf{x}_\beta}\right)\cdot\mathbf{n}_{\beta\alpha} \approx \frac{\rho(\mathbf{x}_\beta + h_{\beta\alpha}\mathbf{n}_{\beta\alpha},t) - \rho(\mathbf{x}_\beta,t)}{h_{\beta\alpha}}\,. \tag{25}$$

where $h_{\beta\alpha}$ is small increment. Choosing $h_{\beta\alpha} = |\mathbf{x}_\alpha - \mathbf{x}_\beta|$, we obtain $\rho(\mathbf{x}_\beta + h_{\alpha\beta}\mathbf{n}_{\beta\alpha}) = \rho(\mathbf{x}_\alpha)$. The right-hand side of eq. 22 can thus be calculated as

$$\begin{aligned} D\int_{\Omega_\beta}\mathrm{d}\mathbf{x}\;\nabla\cdot\nabla\rho(\mathbf{x},t) &= D\sum_{\alpha\sim\beta}\frac{\rho(\mathbf{x}_\alpha,t) - \rho(\mathbf{x}_\beta,t)}{h_{\beta\alpha}}\mathcal{S}_{\beta\alpha} \\ &= D\sum_{\alpha\sim\beta}\frac{\frac{\rho_\alpha(t)}{\mathcal{V}_\alpha} - \frac{\rho_\beta(t)}{\mathcal{V}_\beta}}{h_{\beta\alpha}}\mathcal{S}_{\beta\alpha} \end{aligned} \tag{26}$$

Combining eq. 22 and eq. 26 yields a master equation (eq. 10)

$$\frac{\mathrm{d}}{\mathrm{d}t}\rho_\beta(t) = D\sum_{\alpha\sim\beta}\frac{1}{h_{\alpha\beta}}\frac{\mathcal{S}_{\alpha\beta}}{\mathcal{V}_\alpha}\rho_\alpha(t) - D\sum_{\alpha\sim\beta}\frac{1}{h_{\beta\alpha}}\frac{\mathcal{S}_{\beta\alpha}}{\mathcal{V}_\beta}\rho_\beta(t) \tag{27}$$

with

$$Q_{\alpha\beta,\text{adjacent}} = D\frac{1}{h_{\alpha\beta}}\frac{\mathcal{S}_{\alpha\beta}}{\mathcal{V}_\alpha} \tag{28}$$

Thus the flux factor for isotropic diffusion [7] is

$$\Phi_{\alpha\beta} = D\frac{1}{h_{\alpha\beta}} = \frac{\sigma^2}{2}\frac{1}{h_{\alpha\beta}}\,. \tag{29}$$

II. WATER MODELS

A. Overview of computational experiments

Number of grid cells: $N_r = 10$, $N_s = 80$, $N_o = 80$, $N_d = 64,000$
Radial grid between 0.2 nm and 0.4 nm.

MD simulation time steps: $N_t = 4 \cdot 10^7$

Speed-up: $N_t/N_d = 625$

	parameters [ps]	eigenvectors	ITS (top-bottom) [ps]	ITS (left-right) [ps]
SqRA, vacuum	$\tau_c = 0.001$	Fig. 1	712	197
	$\tau_c = 0.010$	Fig. 1	71.3	19.6
	$\tau_c = 0.100$	Fig. 1	7.13	1.97
	$\tau_c = 1.000$	Fig. 1	0.71	0.20
MD, vacuum,	$\tau_c = 0.001\,,\ \tau_{\mathrm{MSM}} = 0.1$	Fig. 2	8.43	4.45
$R > 0.41$ nm omitted	$\tau_c = 0.010\,,\ \tau_{\mathrm{MSM}} = 0.1$	Fig. 2	2.03	1.06
	$\tau_c = 0.100\,,\ \tau_{\mathrm{MSM}} = 0.1$	Fig. 2	0.88	0.47
	$\tau_c = 1.000\,,\ \tau_{\mathrm{MSM}} = 0.1$	Fig. 2	0.87	(?)
MD, vacuum,	$\tau_c = 0.001\,,\ \tau_{\mathrm{MSM}} = 0.1$	Fig. 3	8.16	4.43
$R > 0.41$ nm included	$\tau_c = 0.010\,,\ \tau_{\mathrm{MSM}} = 0.1$	Fig. 3	1.97	1.05
	$\tau_c = 0.100\,,\ \tau_{\mathrm{MSM}} = 0.1$	Fig. 3	0.85	0.47
	$\tau_c = 1.000\,,\ \tau_{\mathrm{MSM}} = 0.1$	Fig. 3	0.84	0.45
MD, explicit LJ solvent,	$\tau_c = 1.000\,,\ \tau_{\mathrm{MSM}} = 0.1$	Fig. 4	1.69	0.5
$R > 0.41$ nm omitted	$\tau_c = 10.00\,,\ \tau_{\mathrm{MSM}} = 0.1$	Fig. 4	1.0	(?)
MD, explicit LJ solvent,	$\tau_c = 1.000\,,\ \tau_{\mathrm{MSM}} = 0.1$	Fig. 5	1.57	0.47
$R > 0.41$ nm included	$\tau_c = 10.00\,,\ \tau_{\mathrm{MSM}} = 0.1$	Fig. 5	0.98	0.57

The label (?) means that this eigenvector did not occur among the first five eigenvectors and we therefore cannot report the implied timescale.

B. Grid-based model in vacuum

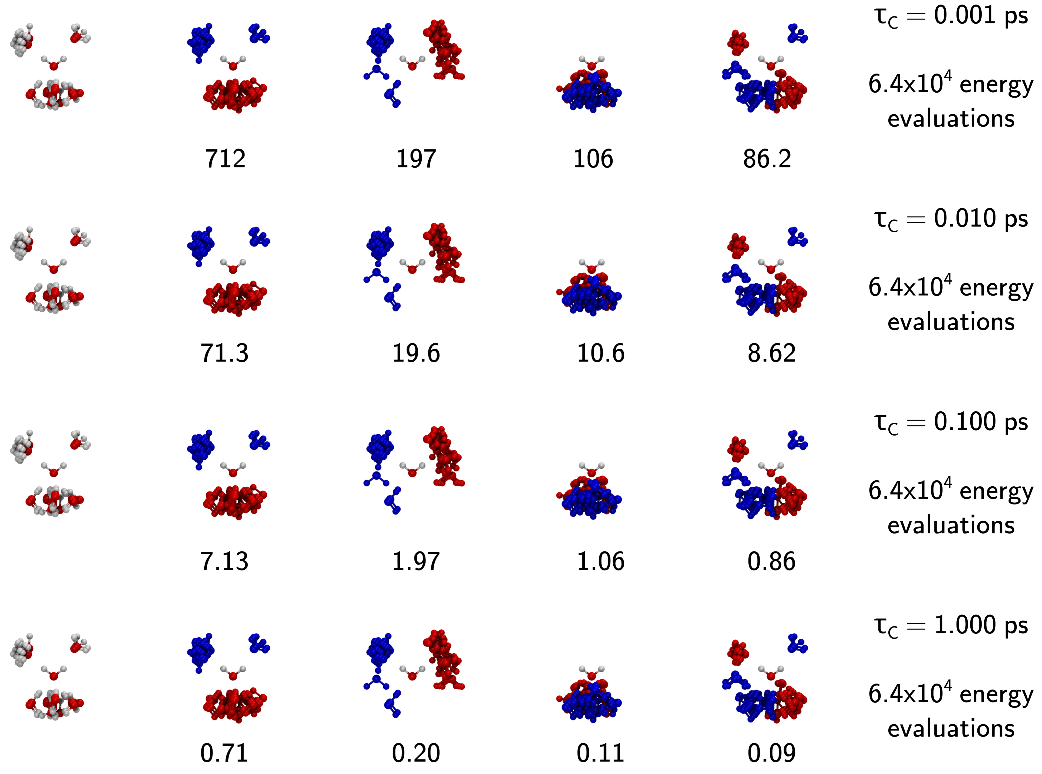

FIG. 1. First five grid-based eigenvectors in vacuum. **Blue**: 30 configurations corresponding to the most negative entries in the eigenvector. **Red**: 30 configurations corresponding to the most positive entries in the eigenvector. For the 1st eigenvector, 30 configurations corresponding to the largest absolute values in the eigenvector are shown. Corresponding implied timescales are noted below each eigenvector.

C. MSM in vacuum

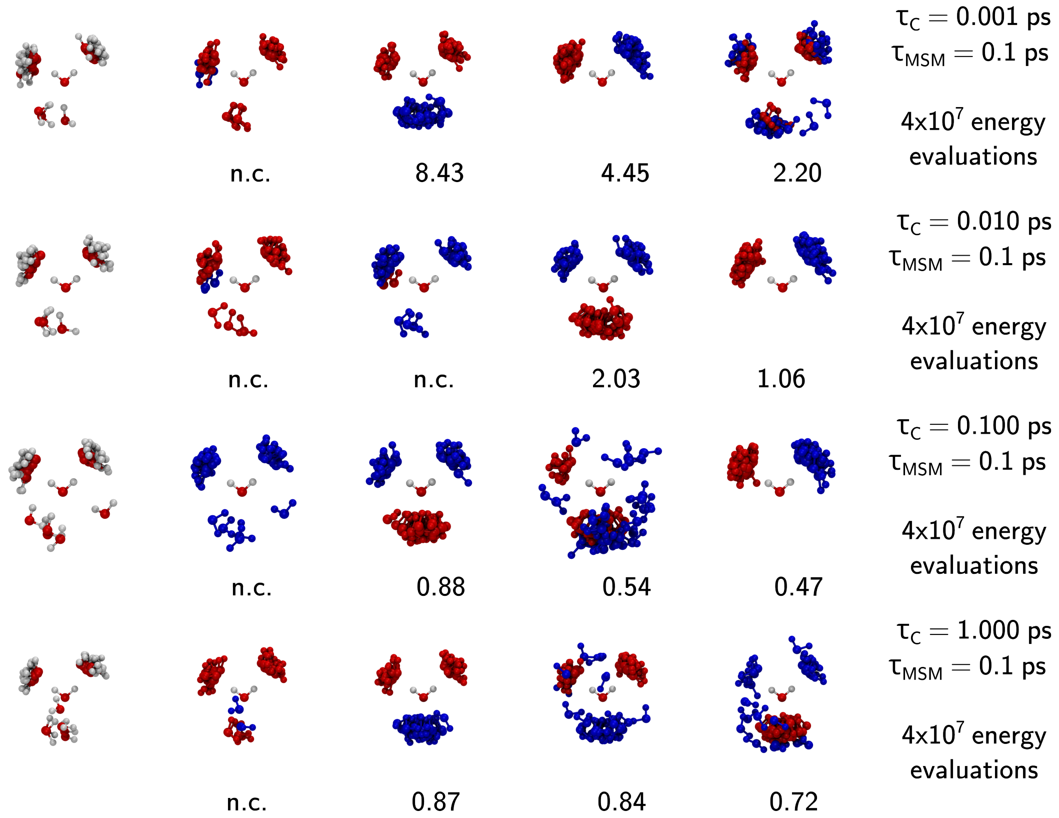

FIG. 2. First five MD-MSM eigenvectors in vacuum; boundary conditions: frames with COM-COM distance > 0.41 nm are omitted. For explanation of blue/red structures see description in Fig. 1. The abbreviation n.c. means (eigenvalue) not converged.

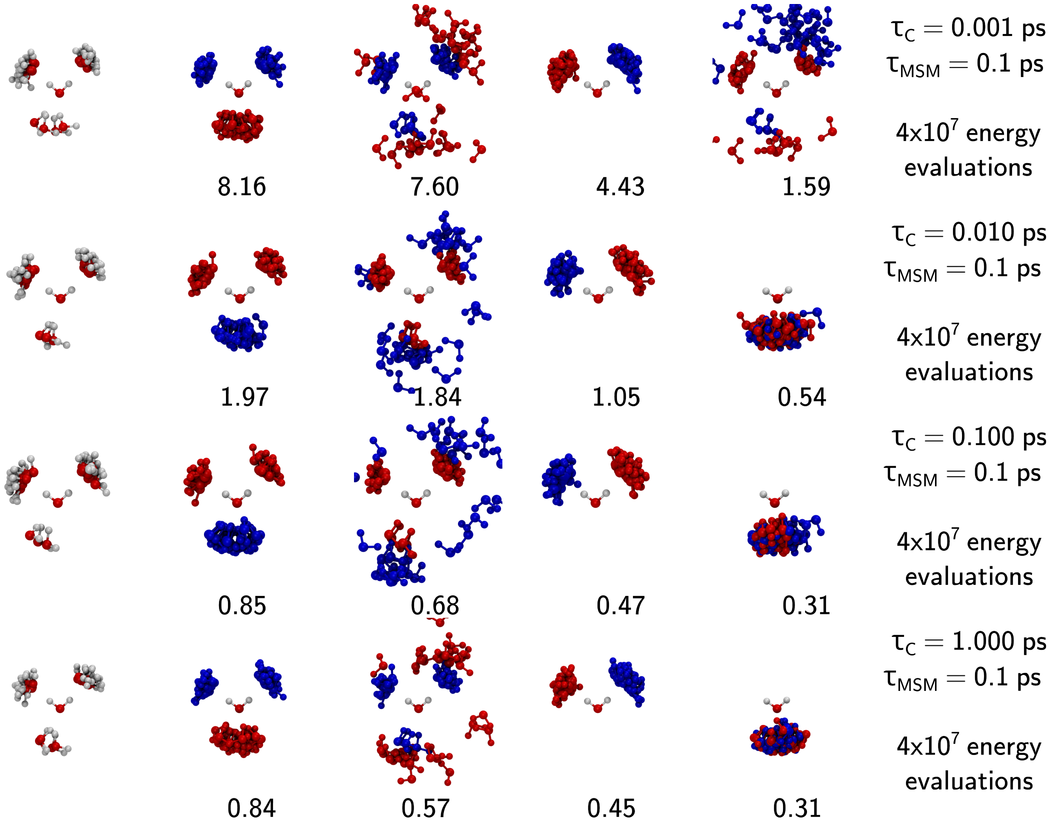

FIG. 3. First five MD-MSM eigenvectors in vacuum; boundary conditions: frames with COM-COM distance > 0.41 nm are assigned to the largest radius. For explanation of blue/red structures see description in Fig. 1.

D. MSM in explicit solvent

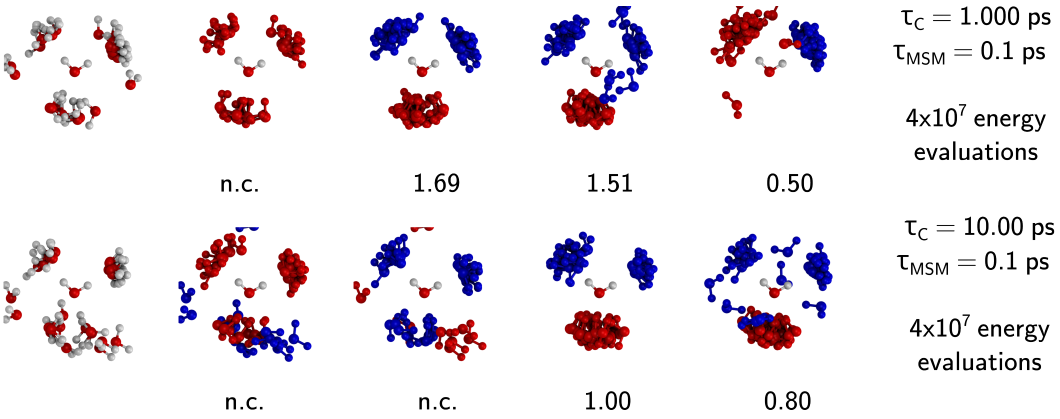

FIG. 4. First five MD-MSM eigenvectors in explicit LJ solvent; boundary conditions: frames with COM-COM distance > 0.41 nm are omitted. For explanation of blue/red structures see description in Fig. 1. The abbreviation n.c. means (eigenvalue) not converged.

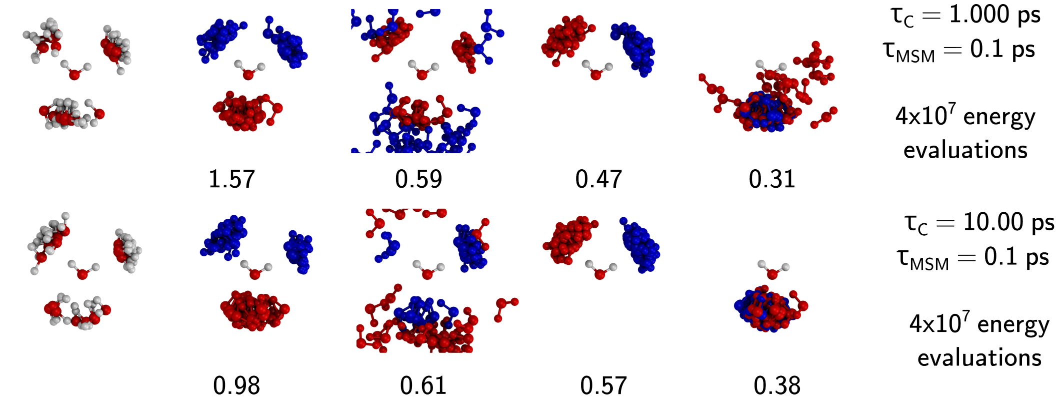

FIG. 5. First five MD-MSM eigenvectors in explicit LJ solvent; boundary conditions: frames with COM-COM distance > 0.41 nm are assigned to the largest radius. For explanation of blue/red structures see description in Fig. 1.

III. LARGER SYSTEMS

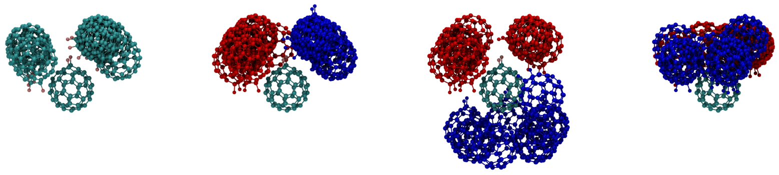

FIG. 6. With molgri-SQRA estimated probability distribution and slowest processes in fluorinated fullerene dimer $C_{60}F_2$-$C_{60}F_2$. The grid used contains $N_o = 80$, $N_s = 80$ and $N_r = 10$ (between 0.8 and 1.3 nm) points.

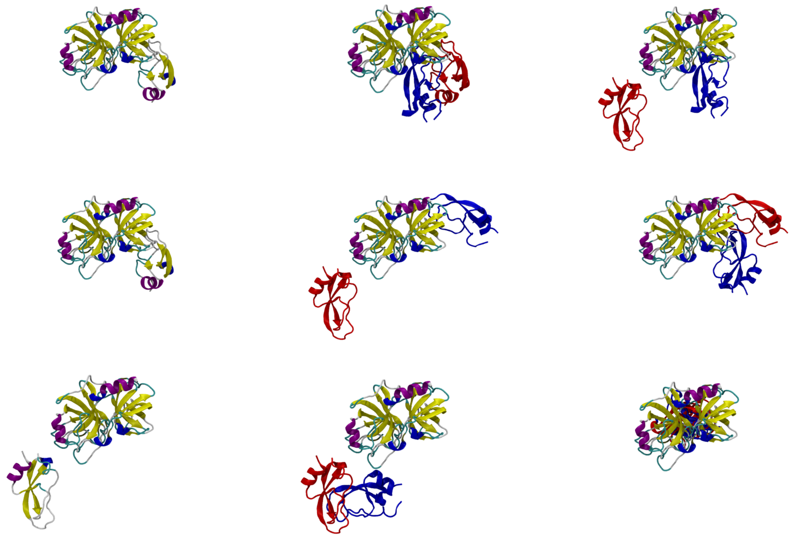

FIG. 7. With molgri-SQRA estimated probability distribution and slowest processes in BPTI-Trypsine complex. A different grid was used in each row. Top: $N_o = 70$, $N_s = 100$ and $N_r = 10$ (between 4.0 and 5.0 nm), middle: $N_o = 100$, $N_s = 70$ and $N_r = 10$ (between 4.0 and 5.0 nm), bottom: $N_o = 80$, $N_s = 80$ and $N_r = 15$ (between 3.5 and 5.0 nm). The poses accounting for at least 40 % of positive and negative probability density flow are shown.

IV. VORONOI TESSELLATION IN CARTESIAN COORDINATES FOR THE TRANSLATIONAL GRID

For a Voronoi grid in Cartesian coordinates, the distances between adjacent translation grid points $\mathbf{t_i}$ and $\mathbf{t_j}$ are Euclidean distances:

$$h_{ij} = \|\mathbf{t_j} - \mathbf{t_i}\| = \sqrt{(\mathbf{t_j} - \mathbf{t_i})^\top \cdot (\mathbf{t_j} - \mathbf{t_i})}\,, \tag{30}$$

where $\mathbf{t}^\top$ denotes the transpose of $\mathbf{t}$. To define the surfaces between neighbouring cells we consider the convex hull around a translation grid point $\mathbf{t_i}$. The plane $\mathscr{P}_{ij}$ defined by the normal vector $\mathbf{t_i} - \mathbf{t_j}$ and the mid-way point $\frac{\mathbf{t_i}+\mathbf{t_j}}{2}$ must contain the division surface S_{ij} since this plane fulfills the Voronoi condition of equal distance to both neighbouring points. The edges of this surface are the intersections of the plane $\mathscr{P}_{ij}$ with other planes $\mathscr{P}_{ik}$ for other neighbouring points $\mathbf{t_k} \sim \mathbf{t_i}$. Thus the surface S_{ij} must have the form of a convex polygon. The area of a triangle with side vectors $\mathbf{a}$ and $\mathbf{b}$ (Fig 8a) can be calculated as:

$$S_{\text{triangle}} = \frac{1}{2}\mathbf{a} \times \mathbf{b}\,. \tag{31}$$

Since every convex polygon can be broken up into triangles as indicated by Fig. 8b, its area can be obtained as the sum of all triangle areas (Shoelace formula [13]). The polygon is defined by the Voronoi vertices shared between $\mathbf{t_i}$ and $\mathbf{t_j}$: $\mathbf{v_0}, \mathbf{v_1} \ldots \mathbf{v_n}$. Given that the vertices are sorted anticlockwise (or clockwise), the area of the polygon with these vertices can be expressed as the sum of triangle areas:

$$S_{ij} = \sum_{k=1}^{n-1} \frac{1}{2}(\mathbf{v_0} - \mathbf{v_k}) \times (\mathbf{v_{k+1}} - \mathbf{v_k})\,. \tag{32}$$

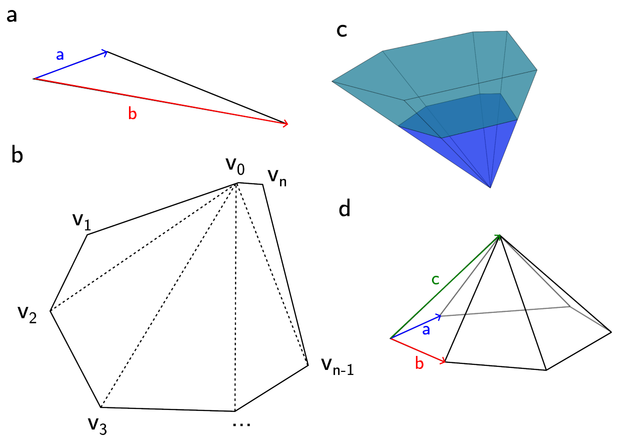

FIG. 8. **a** Triangle with side vectors **a** and **b**. **b** Breaking up any polygon with ordered vertices $\mathbf{v_0}, \mathbf{v_1} \ldots \mathbf{v_n}$ into triangles to calculate its surface via Shoelace formula. **c** Example pyramid (dark blue) and frustum (light blue). **d** An example of the three vectors needed to calculate the volume of a pyramid (**a** and **b** on a base, **c** on a side).

Finally, the volumes of the convex hulls are of interest to us. Since the planes separating points that differ only in their radii are parallel, the form of the convex hulls is either a n-sided pyramid (for points at the smallest radius) or a frustum (pyramid truncated with a plane parallel to the base), see examples in Fig. 8c. The volume of a pyramid is $\frac{1}{3}hS$ where h is the height and S the base surface, where the surface is given by eq. 32. The height of the pyramid is most easily obtained from a projection of a side vector ($\mathbf{c}$, see Fig. 8d) onto

a base normal vector which is obtained as the cross product of base vectors **a** and **b**. The height is therefore

$$h_{\text{pyramid}} = \left| \mathbf{c} \cdot \frac{(\mathbf{a} \times \mathbf{b})}{||(\mathbf{a} \times \mathbf{b})||} \right| \tag{33}$$

where $||\cdot||$ represents the length of a vector and $|\cdot|$ the absolute value.

Again assuming that the base polygon of the smallest-radius cell $\mathbf{t_i}$ has vertices labelled $\mathbf{v_0}, \mathbf{v_1} \ldots \mathbf{v_n}$ and that $\mathbf{t_j}$ is the cell above it, we can therefore express the volume as:

$$V_i = \frac{1}{3} \left| \mathbf{v_0} \cdot \frac{((\mathbf{v_1} - \mathbf{v_0}) \times (\mathbf{v_n} - \mathbf{v_0}))}{||((\mathbf{v_1} - \mathbf{v_0}) \times (\mathbf{v_n} - \mathbf{v_0}))||} \right| S_{ij} \,. \tag{34}$$

If the point $\mathbf{t_i}$ is not at the smallest radius, its convex hull is a frustum and its volume can be calculated as the difference in volumes between a pyramid formed by its own vertices and smaller pyramid formed by the vertices of the cell below it. It should be noted that the hulls around points in the outermost shell (largest radii) are not strictly defined. Currently, we position an additional set of voronoi vertices outside the largest set of points as if there was an additional shell of points beyond the last one. However, we will consider boundary conditions more closely in future work.

V. DIFFERENCE BETWEEN VORONOI GRIDS IN SPHERICAL AND CARTESIAN COORDINATES

We consider the relative error in the distance between adjacent cells $h_{\alpha\beta}$

$$\eta_h = \frac{h_{\alpha\beta}^{\text{Cartesian}} - h_{\alpha\beta}^{\text{spherical}}}{h_{\alpha\beta}^{\text{Cartesian}}} \,, \tag{35}$$

the intersecting surface between adjacent cells $\mathcal{S}_{\alpha\beta}$

$$\eta_{\mathcal{S}} = \frac{\mathcal{S}_{\alpha\beta}^{\text{Cartesian}} - \mathcal{S}_{\alpha\beta}^{\text{spherical}}}{\mathcal{S}_{\alpha\beta}^{\text{Cartesian}}} \,, \tag{36}$$

and the volume of a grid cell $\mathcal{V}_\alpha$

$$\eta_{\mathcal{V}} = \frac{\mathcal{V}_{\alpha}^{\text{Cartesian}} - \mathcal{V}_{\alpha}^{\text{spherical}}}{\mathcal{V}_{\alpha}^{\text{Cartesian}}} \,. \tag{37}$$

To gauge whether the spherical Voronoi grid systematically under- or overestimates the geometric parameters of the Cartesian grid, we consider the signed relative error rather than the absolute relative error. Fig. 9 shows the distribution of the relative errors for various grid sizes.

Differences between a Cartesian and spherical Voronoi grid decrease rapidly with the number of directions N_s and average above 10% only for the smallest choice of N_s. The differences are small but not necessarily negligible.

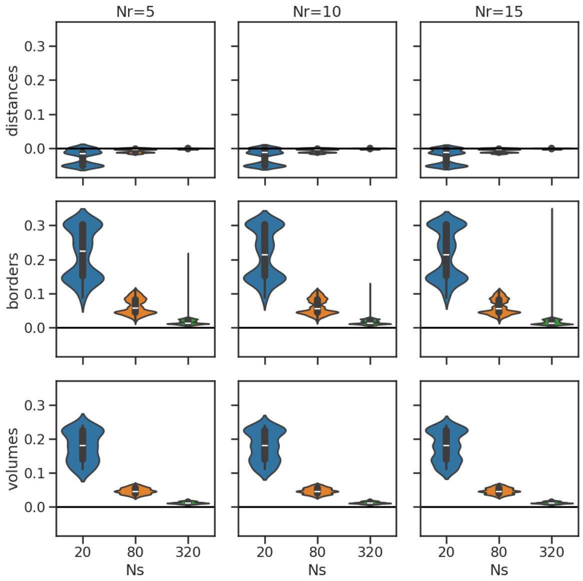

FIG. 9. The violin plots show relative signed errors of Cartesian to spherical grid for $N_o = 1$, N_s=20, 80, 320, and N_r=5, 10, 15 distances between 0.2 and 0.4 nm.

VI. REFERENCES

[1] Wikipedia, "Fokker-planck equation," (2024), https://en.wikipedia.org/wiki/Fokker-Planck_equation [Accessed: 27 Sep 2024].

[2] Wikipedia, "Divergence," (2024), https://en.wikipedia.org/wiki/Divergence [Accessed: 27 Sep 2024].

[3] B. G. Keller and P. G. Bolhuis, "Dynamical reweighting for biased rare event simulations," Annual Review of Physical Chemistry **75**, 137–162 (2024).

[4] Wikipedia, "Master equation," (2024), https://en.wikipedia.org/wiki/Master_equation [Accessed: 27 Sep 2024].

[5] J.-H. Prinz, H. Wu, M. Sarich, B. Keller, M. Senne, M. Held, J. D. Chodera, C. Schütte, and F. Noé, "Markov models of molecular kinetics: Generation and validation," The Journal of chemical physics **134** (2011).

[6] H. C. Lie, K. Fackeldey, and M. Weber, "A square root approximation of transition rates for a markov state model," SIAM Journal on Matrix Analysis and Applications **34**, 738–756 (2013).

[7] L. Donati, M. Heida, B. G. Keller, and M. Weber, "Estimation of the infinitesimal generator by square-root approximation," Journal of Physics: Condensed Matter **30**, 425201 (2018).

[8] M. Heida, M. Kantner, and A. Stephan, "Consistency and convergence for a family of finite volume discretizations of the Fokker–Planck operator," ESAIM: Mathematical Modelling and Numerical Analysis **55**, 3017–3042 (2021).

[9] L. Donati, M. Weber, and B. G. Keller, "Markov models from the square root approximation of the Fokker–Planck equation: Calculating the grid-dependent flux," Journal of Physics: Condensed Matter **33**, 115902 (2021).

[10] Wikipedia, "Divergence theorem," (2024), https://en.wikipedia.org/wiki/Divergence_theorem [Accessed: 27 Sep 2024].

[11] Wikipedia, "Fick's laws of diffusion," (2024), https://en.wikipedia.org/wiki/Fick's_laws_of_diffusion [Accessed: 27 Sep 2024].

[12] Wikipedia, "Directional derivative," (2024), https://en.wikipedia.org/wiki/Directional_derivative [Accessed: 27 Sep 2024].

[13] B. Braden, "The surveyor's area formula," The College Mathematics Journal **17**, 326–337 (1986).
